# Rac1 deficiency impairs postnatal development of the renal papilla

**DOI:** 10.1038/s41598-022-24462-5

**Published:** 2022-11-24

**Authors:** Nobuhiro Ayuzawa, Mitsuhiro Nishimoto, Wakako Kawarazaki, Shigeyoshi Oba, Takeshi Marumo, Atsu Aiba, Toshiro Fujita

**Affiliations:** 1 Division of Clinical Epigenetics, Research Center for Advanced Science and Technology, The University of Tokyo, 4-6-1 Komaba, Meguro-Ku, Tokyo 153-8904 Japan; 2 Department of Internal Medicine, Division of Nephrology and Hypertension, International University of Health and Welfare Mita Hospital, Minato-Ku, Tokyo Japan; 3 Center for Basic Medical Research at Narita Campus, International University of Health and Welfare, Chiba, Japan; 4 Laboratory of Animal Resources, Center for Disease Biology and Integrative Medicine, Graduate School of Medicine, The University of Tokyo, Bunkyo-Ku, Tokyo Japan; 5 Shinshu University School of Medicine, Nagano, Japan; 6 Research Center for Social Systems, Shinshu University, Nagano, Japan

**Keywords:** Kidney, Cell signalling

## Abstract

Development of the renal medulla continues after birth to form mature renal papilla and obtain urine-concentrating ability. Here, we found that a small GTPase, Rac1, plays a critical role in the postnatal development of renal papilla. Mice with distal tubule-specific deletion of Rac1 reached adulthood but showed polydipsia and polyuria with an impaired ability to concentrate urine. The elongation of renal papilla that occurs in the first weeks after birth was impaired in the Rac1-deficient infants, resulting in shortening and damage of the renal papilla. Moreover, the osmoprotective signaling mediated by nuclear factor of activated T cells 5, which is a key molecule of osmotic response to osmotic stress in renal medulla, was significantly impaired in the kidneys of the Rac1-deficient infants. These results demonstrate that Rac1 plays an important role in the development of renal papilla in the postnatal period, and suggested a potential link between Rac1 and osmotic response.

## Introduction

Kidney development occurs during fetal life and continues after birth. In rodents, the renal medulla is formed during the last few days of fetal life and continues to grow with elongation of the distal tubules until several weeks after birth to form mature renal papilla^1^. The development of renal medulla is necessary for the acquisition of urine-concentrating capacity, which occurs after birth^2–4^ to adapt to the extra-maternal environment. It has been suggested that various stimuli and molecules are involved in the development of renal medulla^1^. The deficiency of genes including *Wnt7b*^5^, *Fgf7*^6^, *Fgfr2*^7^, *Egfr*^8^, *Agt*^9,10^, *Ace*^11^, *Agtr1a/b*^12^, *Bmp4*^13^, *Alk3*^14^, *Adamts1*^15^, *Brn1*^16^, and *Esrrg*^17^ leads to defect or hypoplasia of renal medulla in mice. In addition, some researchers have indicated that an appropriate osmotic response against increasing osmotic stress in the renal medulla after birth is required for the postnatal development of renal medulla^18^. Deficiency of nuclear factor of activated T cells 5 (NFAT5), which is a key molecule in the osmotic response^19^, leads to the shortening and damage of renal papilla, resulting in severe impairment of urine-concentrating ability^20^.

Rac1, a member of the Rho family of GTPases, is involved in various biological processes, such as reorganization of the actin cytoskeleton in the regulation of cell morphology and motility^21–23^, cell polarity^24,25^, generation of reactive oxygen species^26^, cell cycle progression^27^, and modulation of the activity of transcription factors^28,29^, including NFAT5^30^. Reflecting the essential role of Rac1 in diverse cell signaling, its involvement in developmental processes has been reported in some tissues including neurons^31,32^, lens^33^, lymphatic vessels^34^, cartilage^35^, and limbs^36^. Given that Cdc42, another member of the Rho GTPases that coordinate with Rac1 to regulate cell motility or polarity, plays a critical role in the development of distal renal tubules^37,38^, the involvement of Rac1 in renal tubular development is reasonable. However, the role of Rac1 in the development of distal tubules is not yet fully clarified. Here, we generated mice with distal tubule-specific deletion of Rac1 and observed that they showed shortening and damage of renal papilla, which resulted in the impairment of urine-concentrating ability. In addition, we found that the osmotic response mediated by NFAT5, which is necessary for the appropriate development of renal papilla in the postnatal period, was reduced in the distal tubule-specific Rac1 deficient mice.

## Methods

### Animals

All animal procedures were approved by Institutional Animal Care and Use Committee of The University of Tokyo and performed according to the ARRIVE guidelines. All methods were performed in accordance with the relevant guidelines and regulations. Distal tubule-specific Rac1-deficient mice were generated by crossing *Rac1*-floxed mice^32^ on a C57BL/6 J background with transgenic mice expressing Cre recombinase under control of the Ksp-cadherin promoter (*Ksp1.3-cre*)^39^ on a C57BL/6 J background. In the *Rac1*-floxed mice, the *loxP* sites were inserted to delete the entire exon 1 of the *Rac1* gene, as previously described^32^. *Ksp1.3-cre* mice were purchased from the Jackson Laboratory. The adult mice used in the experiments were male littermates between 6 and 9 weeks of age. We used neonatal littermates at 2 and 14 days of age and of both sexes in some experiments.

### Water deprivation and urinalysis

Mice were caged in individual metabolic cages (Natsume) with water provided ad libitum for 24 h to collect urine in a steady state. Then, we started 36 h of water deprivation, with measurement of body weight just before and after the deprivation. Urine was collected between 12 and 36 h after the start of deprivation. Urine osmolality was determined by measurement of the freezing point depression in a laboratory at SRL Co.

### Histomorphometric analysis

Kidneys fixed in 4% paraformaldehyde solution were embedded in paraffin, and cross sections (3 μm) were stained with hematoxylin and eosin or Masson’s trichrome in a laboratory at Histological Research Institute, Inc. Images were digitally captured using a Leica DMI4000B microscope and the Leica Application Suite software (Leica). In some sections stained with Masson’s trichrome, the fibrosis fraction was determined by computerized pixel counting using Image J software (National Institutes of Health). For the morphometric analysis of renal papilla, coronal sections just flanking the papilla were digitally captured, and the length of papilla and thickness of cortex were measured using Image J software.

### Quantitative RT-PCR

Total RNA was extracted with the RNeasy Mini Kit (Qiagen) and converted into complementary DNA via the High-Capacity cDNA Reverse Transcription Kit (Applied Biosystems). Quantitative PCR was performed on a StepOnePlus Real-Time PCR System (Applied Biosystems), using a pre-designed primer–probe set (TaqMan Gene Expression Assay, Mm01201653_mH, Applied Biosystems) with TaqMan Gene Expression Master Mix (Applied Biosystems) for the *Rac1* gene, and primers listed in Supplementary Table S1 with Power SYBR Green PCR Master Mix (Applied Biosystems) for the other genes.

### Immunoblot analysis

We performed immunoblot analysis as previously described with some modifications^40^. Protein lysates of kidney samples or cultured cells were prepared using RIPA buffer [50 mM Tris-HCl (pH 7.4), 150 mM NaCl, 1% IGEPAL CA-630, 0.5% sodium deoxycholate, 0.1% SDS] or a urea-containing buffer [50 mM Tris-HCl (pH 7.4), 150 mM NaCl, 4 M urea, 1% SDS, 1 mM EDTA], which were supplemented with protease inhibitor cocktail (cOmplete, Roche) and phosphatase inhibitors (50 mM NaF, 10 mM sodium pyrophosphate, 1 mM sodium orthovanadate). Equal amounts of protein were mixed with Laemmli sample buffer supplemented with 2-mercaptoethanol and were incubated at room temperature for 30 min or boiled at 95 °C for 5 min. Each sample was resolved by SDS-PAGE and transferred to polyvinylidene difluoride membranes. The membranes were incubated with primary antibodies overnight at 4 °C, followed by incubation with the corresponding HRP-conjugated secondary antibodies. Signals were detected and scanned with the ECL Prime Western Blotting Detection Reagent (GE Healthcare) and ImageQuant LAS4000 Mini (GE Healthcare), respectively. We used antibodies against Rac1 (23A8, Millipore), GAPDH (sc-32233, Santa Cruz), NKCC2 (AB3562P, Millipore), AQP2 (sc-9882, Santa Cruz), E-cadherin (#3195, Cell Signaling), and NFAT5 (PA1-023, Thermo Fisher).

### Immunofluorescent staining

We performed immunofluorescent staining as previously described^40^ with some modifications. Dewaxed paraffin-embedded sections (3 μm thick) or cryosections (6 μm thick) fixed with 4% paraformaldehyde were boiled in citrate buffer (pH 6.0) for antigen retrieval. Immunostaining of mouse kidney sections with a mouse primary antibody was performed using a mouse-on-mouse immunodetection kit (Vector Laboratories) in combination with Alexa Fluor 488- or Cy3-conjugated streptavidin (Jackson ImmunoResearch). In immunostaining with the other primary antibodies, the sections were blocked with 5% normal donkey serum, and incubated with a primary antibody followed by incubation with the corresponding secondary donkey antibody conjugated with Alexa Fluor 555 (Invitrogen). Double immunostaining was performed by repeating the process of blocking and subsequent incubation with primary and secondary antibodies. Images were digitally captured using a Leica DMI4000B microscope and the Leica Application Suite software (Leica) and were processed for figure preparation in Image J software (NIH). The primary antibodies used included those for Rac1 (610650, BD Biosciences), α-SMA (M0851, Dako), AQP2 (sc-9882, Santa Cruz), THP (sc-19554, Santa Cruz), and E-cadherin (#3195, Cell Signaling). DAPI (D523, Dojindo) was used for counterstaining of nuclei.

### Cell culture and siRNA transfection

mIMCD3 cells, purchased from ATCC, were incubated in Dulbecco’s Modified Eagle’s Medium:Nutrient Mixture F-12 (Thermo Fisher) supplemented with 5% FBS in 5% CO_2_ at 37 °C. Initial osmolality of the medium was around 300 mOsm/kg. In the experiment involving hyperosmotic stress, NaCl or sorbitol was added to increase the osmolality of the medium by 150 mOsm/kg. Transfection of the mouse Rac1 siRNAs (Stealth RNAi, Rac1MSS237708, Thermo Fisher; and Silencer Select RNAi, s72647, Thermo Fisher) and the corresponding scrambled siRNAs (Stealth RNAi siRNA Negative Control Hi GC, Thermo Fisher; and Silencer Negative Control No. 1 siRNA, Thermo Fisher) was performed with Lipofectamine RNAiMAX (Thermo Fisher) using a reverse transfection protocol.

### Rac1 activation assay

We assessed the activity of Rac1 with commercially available kits as previously described^29^. mIMCD3 cells were cultured in medium supplemented with 1% FBS for 24 h and then exposed to hyperosmotic stress. Protein lysates of cells were prepared using a magnesium lysis buffer [25 mM HEPES (pH7.5), 150 mM NaCl, 10 mM MgCl_2_, 1% IGEPAL CA-630, 10% glycerol, 25 mM NaF, 1 mM sodium orthovanadate] supplemented with protease inhibitor cocktail (cOmplete, Roche). Equal amounts of protein were incubated with glutathione beads coupled with glutathione S-transferase fusion protein corresponding to the p21-binding domain of human PAK1 (Upstate) at 4 °C for 60 min. The beads were washed 3 times with the magnesium buffer, resuspended in Laemmli sample buffer supplemented with 2-mercaptoethanol, and were boiled at 95 °C for 5 min to extract proteins bound to the beads. We determined the Rac1 content in these samples by immunoblotting. All gels shown are consecutive portions of the same gel, and there is no grouping of different portions or portions cut from different gels.

### Statistical analysis

All statistical analyses were performed in JMP software (version Pro 13; SAS Institute Inc.). Data are expressed as mean ± SEM, unless specified otherwise. For comparisons between two groups, we used unpaired *t* test for normally distributed data and Wilcoxon’s rank sum test for non-normally distributed data. For multiple comparisons, we performed one-way ANOVA with Tukey’s HSD post hoc test. Two-way repeated-measures ANOVA with Holm-Sidak’s post hoc test was used to evaluate the changes in urine osmolality between before and after dehydration treatment in the two groups. Correlation analysis was performed using Pearson’s correlation coefficients. Data with a *P* value < 0.05 were considered statistically significant. In all experiments, the *n* value represents the number of individual mice or cultured dishes receiving a given treatment.

## Results

### Distal tubule-specific Rac1-deficient mice show impaired urine-concentrating ability in adulthood with shortening and damage of renal papilla

**Figure 1:**
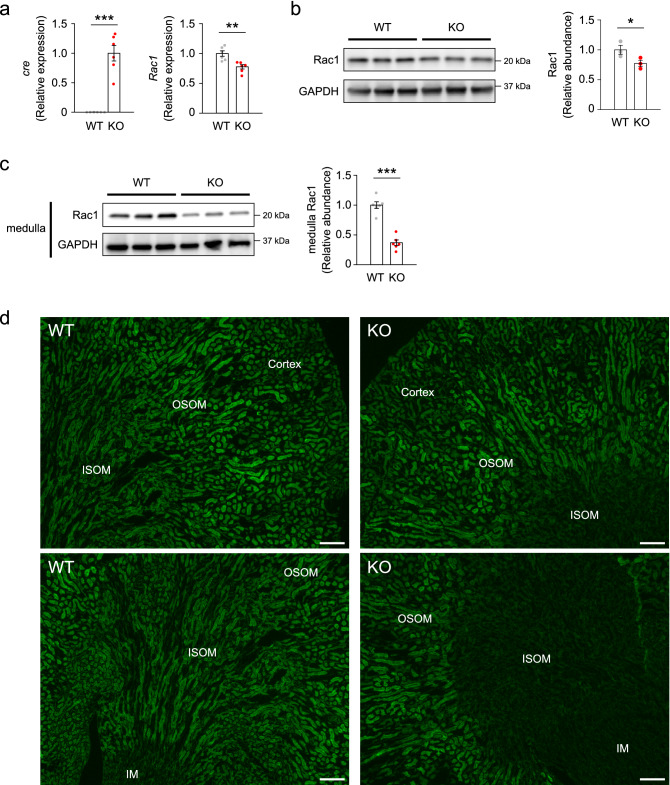
Loss of Rac1 in kidneys of distal tubule-specific Rac1-deficient mice at 6 weeks of age. (**a**) Quantitative analysis of cre and Rac1 gene expression in the whole kidney samples of *Rac1*^flox/flox^ mice (WT) and *Rac1*^flox/flox^;*Ksp1.3-cre*^+*/−*^ mice (KO) (*n* = 6 per group). The expression was normalized to a reference gene (Rps29) and reported relative to the KO group (cre) or WT group (Rac1). (**b**) Representative immunoblots and quantities of Rac1 in the whole kidney samples (*n* = 3 per group). The expression was normalized to a loading control (GAPDH) and reported relative to the WT group. (**c**) Representative immunoblots and quantities of Rac1 in the renal medulla samples (*n* = 6 per group). Renal medulla including papilla, which is composed primarily by the distal tubules and does not include proximal tubules, was cut out under a microscope using a thin razor blade. (**d**) Immunofluorescent staining of Rac1 (green) in the kidneys. Rac1 staining yielded negative results in the renal medulla in KO mice. OSOM: outer stripe of outer medulla, ISOM: inner stripe of outer medulla, IM: inner medulla. Scale bars, 100 μm. Data are expressed as mean ± SEM. Statistical significance was analyzed by unpaired *t* test. *P < 0.01; **P < 0.01; ***P < 0.001.

**Figure 2:**
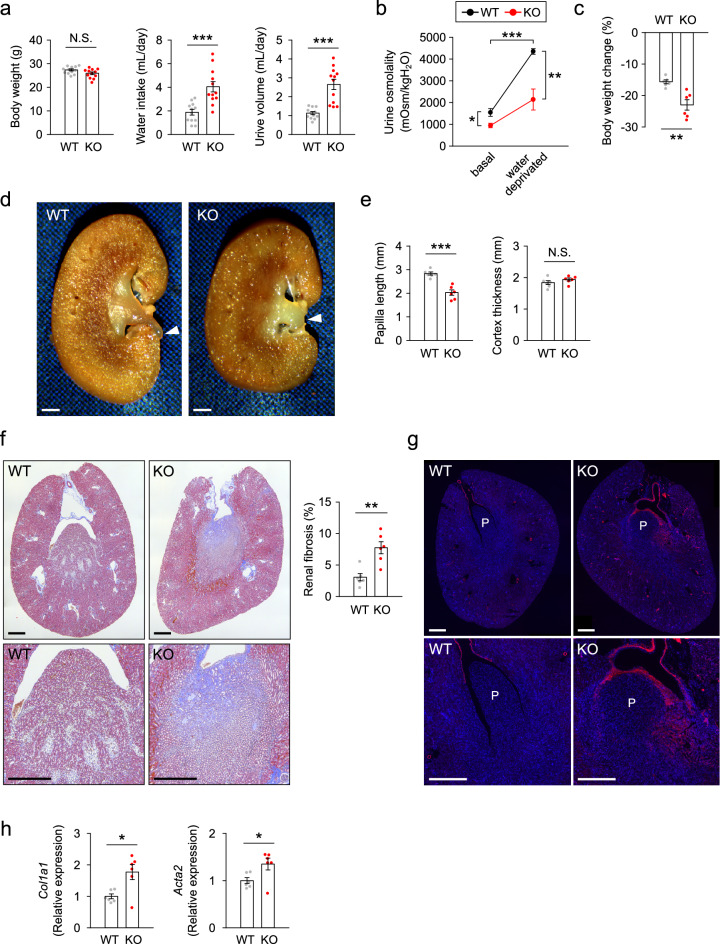
Distal tubule-specific Rac1-deficient mice in adulthood show impaired urine-concentrating ability with shortening and damage of renal papilla. (**a**) Body weight, water intake, and urine volume in WT and KO mice (*n* = 12 per group). (**b**) Urine osmolality at baseline and after water deprivation (*n* = 6 per group). (**c**) Body weight change after the water deprivation (*n* = 6 per group). (**d**) Representative photomicrographs of coronal sections of the kidneys. Arrowheads indicate tips of papilla. The papilla protruded out of the renal hilus in WT mice, but it was clearly shortened in KO mice. (**e**) Quantification of the papilla length and cortex thickness. (**f**) Representative photomicrographs of Masson’s trichrome-stained transverse sections of the kidneys (upper: whole image, lower: enlarged image around papilla) and quantitation of renal fibrosis (*n* = 6 per group). (**g**) Immunofluorescent staining of α-SMA (red) in transverse section of the kidneys. Nuclei were counterstained with DAPI (blue). P: papilla. (**h**) Quantitative analysis of renal expression of fibrosis-associated genes (*n* = 6 per group). The expression was normalized to a reference gene (*Rps29*) and reported relative to the WT group. Data are expressed as mean ± SEM. Statistical significance was analyzed by unpaired *t* test, except in (**b**), and two-way repeated-measures ANOVA with Holm-Sidak’s post hoc test in (**b**). *P < 0.05; **P < 0.01; ***P < 0.001. N.S.: not significant. Scale bars, 1 mm (**d**); 500 μm (**f**, **g**).

**Figure 3:**
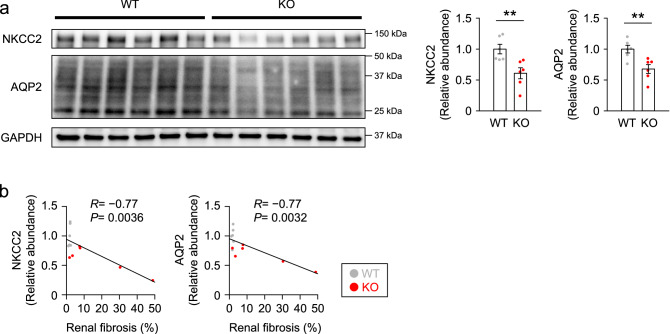
Expression of NKCC2 and AQP2 was reduced in correlation with renal fibrosis in KO mice. (**a**) Immunoblots and quantities of NKCC2 and AQP2 in the kidneys of adult WT and KO mice (*n* = 6 per group). The expression was normalized to GAPDH and reported relative to the WT group. (**b**) Correlation analysis of the expression levels of NKCC2 and AQP2 shown in (**a**) versus renal fibrosis measured by Masson’s trichrome staining. Pearson’s correlation coefficient (*R*) and corresponding significance (*P*) are shown. Data are expressed as mean ± SEM. Statistical significance was analyzed by unpaired *t* test in (**a**). **P < 0.01.

We crossed *Rac1*-floxed mice^32^ with *Ksp1.3*-*cre* mice that express Cre recombinase primarily in all the segment and cell types of distal tubules, from loop of Henle to collecting duct, from the stage of their budding during the embryonic period^39^. We obtained *Rac1*^flox/flox^; *Ksp1.3-cre*^+/−^ mice and their littermate *Rac1*^flox/flox^ mice, which were used as distal tubule-specific Rac1-deficient mice (KO) and wild-type mice (WT), respectively. They were born at expected Mendelian ratios and reached adulthood with no apparent difference in survival rate between the two genotypes. Expression of the *Rac1* gene and protein in the whole kidney samples of KO mice was only slightly reduced compared with the levels in WT mice (Fig. 1a,b), presumably reflecting that the *Ksp1.3-cre*-driven gene recombination occurs with high efficacy in distal tubules that occupy rather a small volume of the entire kidney, but is induced in a small fraction of cells in proximal tubules that occupy a large volume. Indeed, expression of the Rac1 protein in the renal medulla which consists primarily of distal tubules and does not include proximal tubules were significantly decreased in KO mice compared with the levels in WT mice (Fig. 1c). Immunostaining of Rac1 showed the loss of immunolabeling mainly in the medullary region of the kidneys in KO mice (Fig. 1d), confirming successful Rac1 deletion in the distal tubules. In adulthood, the body weight of KO mice was comparable to that of WT mice, but the KO mice showed apparent polydipsia and polyuria (Fig. 2a). In KO mice, the levels of urine osmolality both at baseline and after water deprivation were lower than in WT mice (Fig. 2b). Moreover, KO mice showed significantly greater loss of body weight than WT mice in the water deprivation test (Fig. 2c). Searching for the cause of these phenotypes, we found that the renal papilla of KO mice was shortened and damaged. As shown in Fig. 2d,e, the length of renal papilla in KO mice was shorter than that in WT mice, while the thickness of cortex was comparable between WT and KO mice. Masson’s trichrome staining revealed renal fibrosis, mainly in the medullary region, in KO mice (Fig. 2f). In the immunostaining of α-smooth muscle actin (α-SMA), immunolabeling was found around and inside the renal papilla in KO mice indicating medullary fibrosis (Fig. 2g). Consistent with these observations, the expression of fibrosis-associated genes, *Col1a1* (encoding collagen type I a1) and *Acta2* (encoding α-SMA), was significantly upregulated in the kidneys of KO mice (Fig. 2h). In association with the damage in renal medulla, the abundance of sodium-potassium-chloride cotransporter 2 (NKCC2) and aquaporin 2 (AQP2), which are molecules involved in urine concentration in renal medulla, was reduced in the KO kidneys (Fig. 3a). There was a significant negative correlation between fibrosis and the expression of AQP2 and NKCC2 (Fig. 3b). Collectively, we found that Rac1-deficiency in distal tubules leads to impairment of urine concentrating ability in association with the shortening and damage of renal papilla.

### Rac1 deficiency in distal tubules disrupts elongation of renal papilla during the postnatal period and causes damage

**Figure 4:**
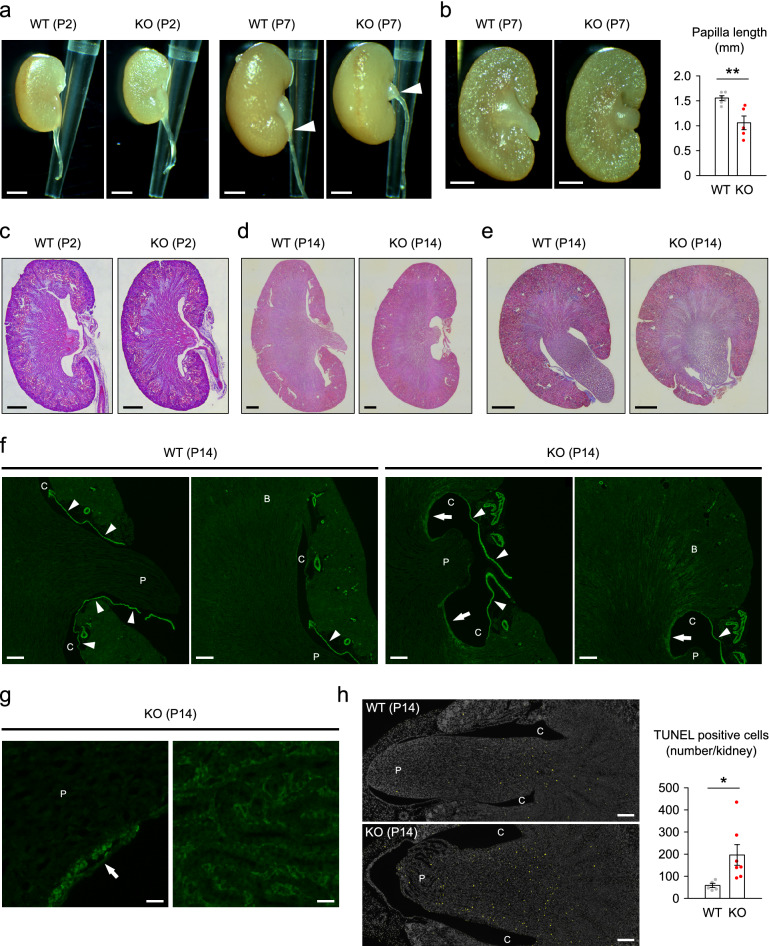
Rac1 deficiency in distal tubules disrupts elongation of renal papilla during postnatal period and causes damage. (**a**) Representative photomicrographs of the kidneys and ureters of WT and KO mice at postnatal day 2 (P2) and day 7 (P7). Arrowheads indicate the tip of papilla. (**b**) Coronal sections of the kidneys and quantification of the papilla length (*n* = 5–6 per group). (**c**, **d**) Coronal sections of the kidneys at P2 (C) and P14 (D) stained with hematoxylin and eosin. (**e**) Transverse sections of the kidneys at P14 stained with Masson’s trichrome. (**f**) Immunofluorescent staining of α-SMA (green) in coronal sections of the kidneys at P14. P: papilla, C: calyces, arrowheads: wall of ureter. In KO mice, slight positive staining for α-SMA is observed in some parts of the wall of the papilla and calyces (arrows) and in some interstitial area at the corticomedullary border (B). (**g**) Magnified image of α-SMA staining. P: papilla. Arrow: α-SMA-positive wall of papilla. Positive staining for α-SMA is observed in the wall of papilla (left) and interstitium of corticomedullary border (right). (**h**) TUNEL (yellow) staining in transverse sections of the kidneys. Nuclei were counterstained with DAPI (gray). P: papilla, C: calyces. The bar graph shows the counts of TUNEL-positive cells in the kidneys (*n* = 5–7 per group). Data are expressed as mean ± SEM. Statistical significance was analyzed by unpaired *t* test. *P < 0.05; **P < 0.01; N.S.: not significant. Scale bars, 1 mm (**a**,**b**); 200 μm (**c**, **f**, **h**); 500 μm (**d**, **e**); 20 μm (**g**).

Given that the development of renal papilla is prominent in the first few weeks after birth in mice, we next analyzed the kidneys of WT and KO mice in this period. The distal tubule-specific deletion of Rac1 in KO mice was confirmed in the postnatal period (Supplementary Fig. S1a–d). The body weight and renal weight were comparable between WT and KO mice at postnatal day 2 (P2) (Supplementary Fig. S2a,b). In WT mice, the renal papilla was small, and their tips were located inside the hilus at P2, but they grew dramatically and extended outside the hilus over the next 2 weeks (Fig. 4a–e). In KO mice, the shape of the renal papilla was similar to that of WT at P2, but the subsequent elongation of the renal papilla was significantly impaired (Fig. 4a–e). The expression of fibrotic genes, *Col1a1* and *Acta2*, in the kidneys did not differ significantly between WT and KO mice at P2 (Supplementary Fig. S2c). Moreover, in immunostaining for α-SMA, the patterns of immunolabeling in the kidneys were similar between WT and KO mice at P2 (Supplementary Fig. S2d). At P14, the gene expression of *Col1a1* and *Acta2* in the kidneys was not statistically different between WT and KO mice, but seemed to be upregulated in some KO mice (Supplementary Fig. S2c), suggesting that fibrosis just start to appear in this period. Actually, we observed that immunolabeling of α-SMA appears in the walls of papilla and in the interstitium of the corticomedullary border in KO mice at P14 (Fig. 4f,g). These results indicate that the renal fibrosis in KO mice begins during the postnatal period. Finally, we found that the TUNEL-positive apoptotic cells were significantly increased in number and located in all areas of the papilla in KO mice (Fig. 4h), while they were localized at the base of the papilla in WT mice, as previously reported^41^. Taking these findings together, the damage of the papilla in KO mice begins during the postnatal period.

### Rac1 deficiency impairs NFAT5-mediated osmoprotective signaling

In exploring the mechanism linking Rac1 deletion and its associated phenotype, we noted that NFAT5-deficient mice reportedly showed shortening of renal papilla and damage at those sites, including cell death and fibrosis^20^, similar to the findings in the mice with distal tubule-specific deficiency of Rac1. NFAT5 is a key molecule of osmotic response, which is upregulated and activated by hyperosmotic stress to induce the transcription of osmoprotective genes^19,42^. In the NFAT5-deficient mice, the expression of osmoprotective genes in response to the hyperosmotic environment in the renal medulla after birth was critically disrupted, resulting in the phenotype mentioned above^20^. Moreover, an in vitro study using a kidney cell line demonstrated that Rac1 is involved in the hyperosmotic stress-induced increase of NFAT5 protein and transcriptional activity of NFAT5^30^. These previous findings led us to hypothesize that the deletion of Rac1 in distal tubules caused shortening and damage of papilla through the impairment of osmotic response mediated by NFAT5.

**Figure 5:**
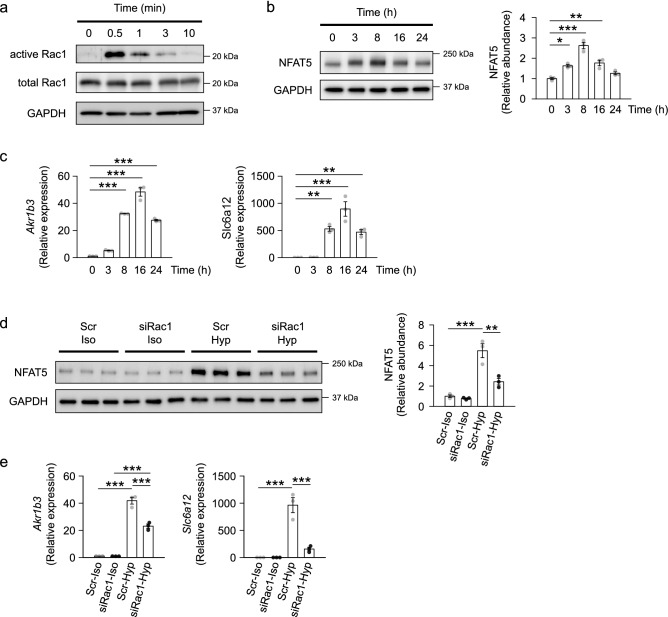
Rac1 deficiency impairs NFAT5-mediated osmoprotective signaling in mIMCD3 cells. (**a**) Hyperosmotic stress induces activation of Rac1 in mIMCD3 cells. mIMCD3 cells were cultured in hyperosmotic medium prepared by adding NaCl for 0, 0.5, 1, 3, or 10 min. Immunoblots of active and total Rac1 are shown. GAPDH was used as loading control. (**b, c**) NFAT5 signaling induced by hyperosmotic stress. mIMCD3 cells were cultured in hyperosmotic medium prepared by adding NaCl for 0, 3, 8, 16, or 24 h. (**b**) Representative immunoblots of NFAT5 (*n* = 3 per group). (**c**) Quantitative analysis of the expression of *Akr1b3* and *Slc6a12* genes, which are osmoprotective genes downstream of NFAT5 (*n* = 3 per group). (**d, e**) Effect of Rac1 knockdown on the hyperosmotic stress-induced NFAT5 signaling. mIMCD3 cells transfected with either scrambled siRNA (Scr) or *Rac1* siRNA (siRac1) were cultured in either isosmotic medium (Iso) or hyperosmotic medium (Hyp) prepared by adding NaCl. (**d**) Immunoblots of NFAT5 after 8 h of isosmotic or hyperosmotic treatment (*n* = 3 per group). (**e**) Quantitative analysis of expression of *Akr1b3* and *Slc6a12* genes after 16 h of isosmotic or hyperosmotic treatment (*n* = 3 per group). The protein and gene expression were normalized to a loading control protein (GAPDH) and a reference gene (*Rps29*), respectively, and reported relative to the value at 0 h or in the Scr-Iso group. Data are expressed as mean ± SEM. Statistical significance was analyzed by one-way ANOVA with Tukey’s HSD post hoc test. *P < 0.05; **P < 0.01; ***P < 0.001.

First, to confirm the in vitro involvement of Rac1 in NFAT5 signaling, we examined the effect of Rac1 deficiency on hyperosmotic stress-induced NFAT5 signaling in in vitro experiments using cultured mouse inner medullary collecting duct cells (mIMCD3). After increasing the osmolarity of the medium by increasing the NaCl concentration, the amount of GTP-bound active Rac1 protein increased significantly with a peak at 0.5 min, indicating that Rac1 was immediately activated (Fig. 5a). This result was consistent with the previous studies using other cell line^43^. The hyperosmotic stress subsequently increased the expression of NFAT5 protein with a peak at 8 h (Fig. 5b), followed by an increase in the gene expression of *Akr1b3* (encoding aldose reductase) and *Slc6a12* (encoding betaine/γ-aminobutyric acid transporter), which are osmoprotective genes downstream of NFAT5, with a peak at 16 h (Fig. 5c). The response of NFAT5 to hyperosmolarity was similarly observed when the osmotic substance was changed from NaCl to sorbitol (Supplementary Fig. S3a). Moreover, knockdown of Rac1 by small interfering RNA (siRNA) significantly suppressed the hyperosmotic stress-induced increase in the expression of NFAT5 protein and osmoprotective genes at 8 h and 16 h, respectively (Fig. 5d,e). We have confirmed that the Rac1 siRNA we used reduces Rac1 expression with sufficient efficiency (Supplementary Fig. S3b,c). These results demonstrated that Rac1 is involved in the hyperosmotic stress-induced increase in NFAT5 protein and subsequent induction of the expression of osmoprotective genes. The same results were obtained with another Rac1 siRNA (data not shown).

**Figure 6:**
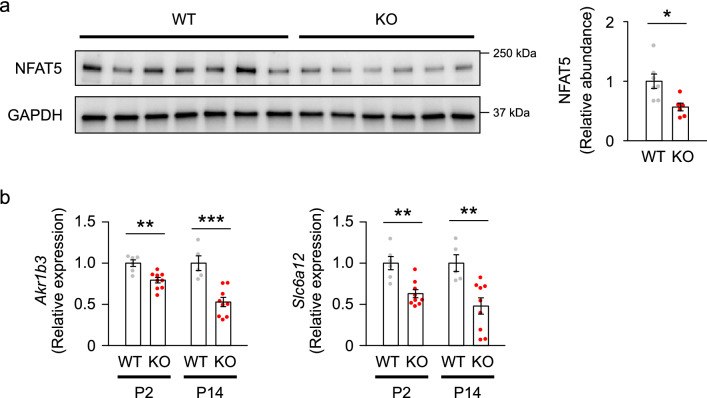
NFAT5 signaling is impaired in the kidneys of KO mice. (**a**) Immunoblots of NFAT5 in the kidneys of WT and KO mice at P14 (*n* = 6–7 per group). (**b**) Quantitative analysis of the expression of *Akr1b3* and *Slc6a12* genes in the kidneys at P2 and P14 (*n* = 5–7 per group). Protein and gene expression was normalized to a loading control protein (GAPDH) and a reference gene (*Rps29*), respectively, and reported relative to the WT group at each time point. Statistical significance was analyzed by unpaired *t* test. *P < 0.05, **P < 0.01, ***P < 0.001.

Given that the osmolarity in renal medulla rises after birth^44,45^, we thought that the Rac1 deletion in KO mice disturbs the NFAT5-mediated signaling in the kidney. Thus, we next analyzed the effect of Rac1 deletion on NFAT5 signaling in the kidneys of KO mice. As shown in Fig. 6a, the renal expression of NFAT5 protein was significantly lower in KO mice than in WT mice at P14. Moreover, KO mice showed reduced expression of *Akr1b3* and *Slc6a12* genes in the kidney compared with WT mice (Fig. 6b). These results suggest that Rac1 deletion impairs postnatal osmoprotective signaling, likely via the key osmotic response regulator NFAT5.

**Figure 7:**
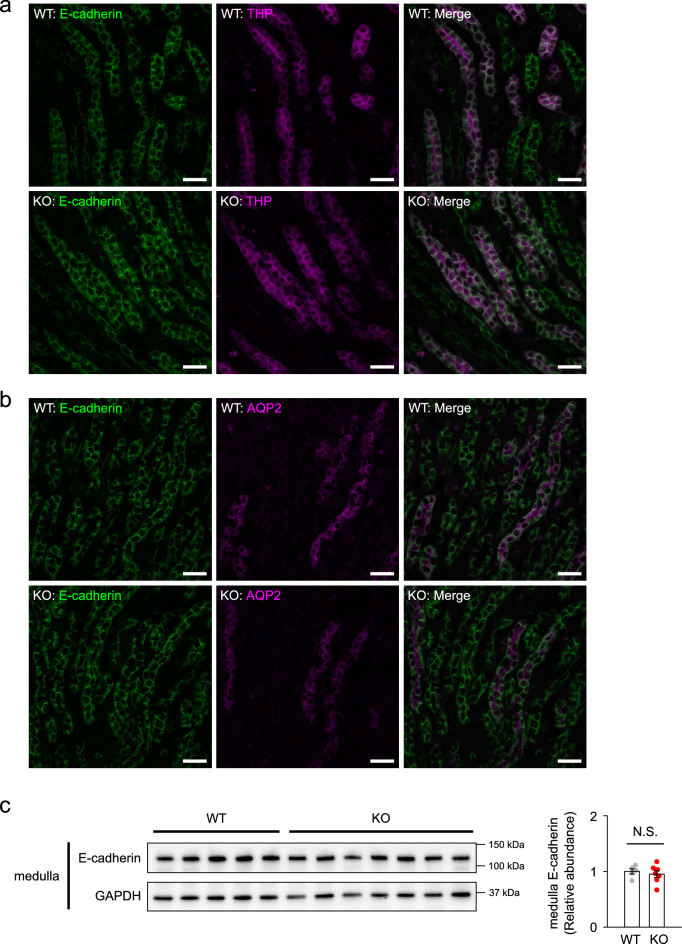
E-cadherin expression in the renal medulla of WT and KO mice at P14. (**a, b**) Immunofluorescent staining of E-cadherin (green) with counterstaining of Tamm-Horsfall protein (THP) (magenta) for thick ascending limbs of loop of Henle (**a**) and AQP2 (magenta) for collecting ducts (**b**) in the renal medulla at P14. Scale bars, 50 μm. (**c**) Immunoblots and quantities of E-cadherin in the renal medulla at P14 (n = 5–7 per group). The expression was normalized to a loading control (GAPDH) and reported relative to the WT group. Data are expressed as mean ± SEM. Statistical significance was analyzed by unpaired *t* test. N.S.: not significant.

Since Rac1 is known to be involved in maintenance of epithelial integrity, we finally examined whether epithelial integrity is disrupted in the medullary tubules of KO mice at postnatal period. Immunostaining patterns of E-cadherin in the thick ascending limbs and collecting ducts in renal medulla at P14 were not different between WT and KO mice (Fig. 7a,b). We observed no difference in the expression of E-cadherin protein in the renal medulla of WT and KO mice at P14 (Fig. 7c).

## Discussion

In the present study, mice with Rac1 deficiency specifically in the distal tubule showed normal renal morphology soon after birth, but exhibited impaired growth of renal papilla and damage in the medullary region during the next few weeks. These observations indicate that Rac1 is involved in some biological signaling that is activated after birth and indispensable for the appropriate development of renal papilla. Our data also suggested a potential involvement of Rac1 in NFAT5-mediated osmoprotective signaling. NFAT5 is a transcription factor that is upregulated and activated upon hyperosmotic stress^46–48^ and binds to osmotic response elements located in the regulatory region of osmotic responsive genes^46,47,49–52^. In the kidney, NFAT5 is highly expressed in the medulla^53^, where the extracellular osmolality becomes extremely high after birth^44,45^ and induces the expression of osmoprotective genes in the medullary region^20,54,55^. This expression in turn causes the intracellular accumulation of organic osmolytes^56,57^, which act to resist the extracellular hyperosmolality. NFAT5-deficient mice reportedly lacked the expression of osmoprotective genes in renal medulla and exhibited impaired growth of renal papilla and damage at those sites^20^, demonstrating that NFAT5-mediated osmoprotective signaling is indispensable for the normal development of renal medulla. It has been demonstrated that Rac1 is activated by hyperosmotic stress and contributes to an osmosensing mechanism forming a complex with osmosensing scaffold for MEKK3 (OSM)^43^. Zhou et al.^30^ demonstrated that the knockdown of Rac1 reduced the hyperosmotic stress-induced transcriptional activity of NFAT5 measured in a renal cell line that expressed a luciferase reporter containing osmotic response elements, suppressing the abundance of NFAT5 protein and transactivation of NFAT5. They also showed that the overexpression of constitutively active and dominant negative mutants of Rac1 increased and decreased the transcriptional activity of NFAT5, respectively^30^. In our study, Rac1 knockdown suppressed the hyperosmotic stress-induced increase of NFAT5 protein and the upregulation of NFAT5 downstream osmoprotective genes, confirming that Rac1 is involved in the osmotic response mediated by NFAT5. Of note, the Rac1 activation and following NFAT5 signaling was only transient. Some adaptive responses or negative feedback that is specific to in vitro condition might be involved in this phenomenon. The slight decrease in the expression of total Rac1 expression after several hours of hyperosmotic stimuli might also be the result of negative feedback induced by preceding activation of Rac1 function. Nevertheless, we observed that the distal tubule-specific Rac1-deficient mice showed the reduced expression of NFAT5 protein and NFAT5 downstream osmoprotective genes in the kidney. These observations suggest a potential involvement of Rac1 in NFAT5 signaling in response to hyperosmotic stress, although causal link between Rac1 and NFAT5 signaling in vivo should be further investigated.

Notably, Rac1 only made a partial contribution to the process of activation of NFAT5 by hyperosmotic stress in the kidney. We observed that both the in vitro Rac1 knockdown in renal cells exposed to hyperosmotic stress and the in vivo Rac1 deletion in distal tubules significantly suppressed the expression of NFAT5 protein, but did not completely abolish its expression or that of its downstream genes. These results were in line with a previous report describing that the knockdown of Rac1 in a renal cell line significantly, but only partially, suppressed the expression of NFAT5 protein and NFAT5-dependent transcriptional activity^30^. Moreover, it was demonstrated that Rac1 knockdown did not affect the hyperosmotic stress-induced nuclear transport of NFAT5^30^, which is a fundamental mechanism of NFAT5 activation. Reflecting the partial role of Rac1 in NFAT5 activation, the phenotype of the distal tubule-specific Rac1-deficient mice was milder than that of NFAT5-deficient mice. NFAT5-deficient mice showed severe impairment of urine-concentrating ability and a substantial proportion of them died after birth^20^, but the distal tubule-specific Rac1-deficient mice rarely died after birth and reached adulthood showing only moderately impaired ability to concentrate urine.

Rac1 is reportedly involved in cell motility^22,23^ and polarity^24,25^ like other Rho GTPases including Cdc42^23,25,58^, and therefore the disruption of Rac1-mediated cell motility and polarity may also contribute to the phenotype of the distal tubule-specific Rac1-deficient mice. Several studies have demonstrated that Rac1 inactivation disrupted lumen formation of cultured renal epithelial cells. However, we observed neither luminal obstruction nor cystogenesis in the distal-tubule-specific Rac1-deficient mice, which differed from the observation in mice with Cdc42 deletion in distal tubules or ureteric buds that showed cystogenesis and obstruction in distal tubules^37,38^. Studies have also shown that Rac1 deficiency impairs the collective migration or elongation of some cultured cells^59^ or embryonic structure^60^ by disturbing cell migration. However, it was unclear why the formation of renal medulla in the embryonic stage was not impaired in the distal tubule-specific Rac1-deficient mice. Recently, Bock et al*.*^61^ demonstrated that mice with uretic bud-specific Rac1-deletion by *Hoxb7*-cre show only mild phenotype at birth, but exhibit renal fibrosis and impairment of urine concentrating ability at 6 months of age. They also found that epithelial integrity and polarity of collecting ducts was normal at birth but disrupted in the 6-month-old uretic-bud-specific Rac1-deficient mice, which could contribute to the development of the phenotype. However, we could not detect clear abnormalities of epithelial integrity in the renal medullary tubules in our distal-tubule-specific Rac1-deficient mice during postnatal period. Meanwhile, our distal-tubule-specific Rac1-deficient mice showed impaired osmoprotective signaling during postnatal period, when the shortening and damage of papilla start. Considering that Rac1 is involved in various cellular signaling at different time, and that there are some differences between the timing and target site of gene recombination by *Hoxb7-cre* and *Ksp1.3-cre* (*Hoxb7-cre* starts recombination at E9.5 and finally targets collecting duct^62^; *Ksp1.3-cre* starts recombination at E15.5 or earlier and finally targets all segment of distal tubules from loops of Henle to collecting ducts, and small population of cells in proximal tubules^39,63^), the result of our study does not necessarily conflict with that of the study of uretic-bud-specific Rac1-deficient mice. Rather, our study and that of uretic bud-specific Rac1 deficient mice are complementary, suggesting that Rac1 plays multiple roles in the distal tubules after birth. Of note, our study could not identify the responsible segment and cell-type of distal tubules for the phenotype of the distal-tubule-specific Rac1-deficient mice. However, given that the phenotype was predominant in renal medulla despite the *Ksp1.3-cre* induces recombination in both cortical and medullary segments of distal tubules, our result suggested that Rac1 might play roles in the medullary distal tubules. Considering the similarity between the phenotype of the uretic-bud- and distal-tubule-specific Rac1-deficient mice, the role of Rac1 in collecting ducts might be unquestionable, but potential roles of Rac1 in thick and thin loop of Henle should also be examined in future study. As for the reason that the phonotype of distal-tubule-specific Rac1-deficent mice arise after birth, because the osmolarity of medulla increase significantly after birth, the effect of the loss of Rac1-NAFT5 pathway might became apparent in the postnatal period. However, it is also possible that the phenotype is due to the timing of recombination by *Ksp1.3-cre* that occurs mostly in late embryogenesis.

We assume that multiple factors are involved in the cause of impaired urine concentrating ability observed in distal tubule-specific Rac1-deficient mice. First, simply, the shortening of medullary thick ascending loop of Henle and collecting ducts, which are responsible for formation of medullary osmotic gradient and water reabsorption, would contribute to the defect in urinary concentration. In addition, the global malfunctioning of cells induced by the cytotoxicity caused by intolerance to hyperosmolarity or disruption of cell polarity and cytoskeleton would cause malfunction of the distal tubules. The reduced expression of NKCC2 and AQP2 would have impaired formation of medullary osmotic gradient and water reabsorption, respectively. Fibrosis in renal medulla might also contribute to impaired urine concentration by disrupting the structure of renal medulla and impairing cellular function of nearby distal tubules. Of note, the damage in renal medulla would impair the formation of medullary osmotic gradient, and in turn, could contribute to the reduction of NFAT5-mediated osmoprotective signaling in the distal-tubule-specific Rac1 deficient mice. We could not detect difference in the renal expression of NKCC2 protein between WT and KO mice at P2 (Supplementary Fig. S4), however, it remains possible that some cellular dysfunction due to Rac1 deficiency may have disrupted the function of NKCC2, which lead to the suppression of NFAT5 signaling observed at this period.

Additionally, our findings do not rule out the potential involvement of Rac1 in other signaling pathways that are essential for the development of renal medulla. Some in vitro studies have indicated that Rac1 could mediate Wnt-^28^, fibroblast growth factor (FGF)-^64^, or bone morphogenetic protein (BMP)-induced signaling^65^. However, while the deficiency of *Wnt7b*^5^, *Fgf7*^6^, *Fgfr2*^7^, *Bmp4*^13^, or *Alk3*^14^ resulted in the impairment of medullary development already in the fetal stage, the Rac1 deletion in distal tubules was not associated with any apparent abnormalities in the renal morphology at birth. Thus, we assumed that the contribution of Rac1 to such signaling in medullary development would be limited, at least during the fetal stage. Rac1 has also been reported to be involved in epidermal growth factor receptor (EGFR)-related signaling^66,67^. Zhang et al.^8^ reported that *Egfr* deletion in ureteric buds of mice leads to thinning of the renal medulla and moderate impairment of the urine-concentrating ability in adulthood. Given that EGFR undergoes crosstalk with angiotensin II receptor type 1 (AGTR1) signaling^67–69^, which is involved in the elongation of renal papilla inducing *Wnt7b* gene expression^70^, and that the mouse phenotypes of *Egfr* deletion^8^ partially overlap with those of *Agtr1a/b* deletion^71^ and *Wnt7b* deletion^5^, it was proposed that EGFR cooperates with AGTR1 and WNT7B during the development of renal medulla. Considering that Rac1 was shown to be involved in EGFR–AGTR1 crosstalk in some studies^67^, alteration in the EGFR signaling could contribute to part of the phenotype of the distal tubule-specific Rac1-deficient mice. Although we could not detect suppression of *Wnt7b* gene expression in the kidneys of the distal tubule-specific Rac1-deficient mice (Supplementary Fig. S2e), it remains possible that Rac1 participates in the EGFR–AGTR1 crosstalk without affecting WNT7B induction.

Congenital anomalies of the kidney and urinary tract (CAKUT) are a common malformation, affecting 0.3–0.6% of newborns^72^ and the leading cause of pediatric renal failure. More than 50 monogenic CAKUT types have been reported to date^72,73^, but only 20% of CAKUT cases are due to these known genetic mutations^74,75^. Medullary dysplasia is a major form of CAKUT. While the atrophy of renal papilla often occurs secondary to urinary obstruction and hydronephrosis, a primary defect in the developmental process of renal medulla leads to the formation of atrophic papilla in some cases. However, the developmental process of renal medulla in the postnatal period remains less understood than that in the prenatal period. Here, we demonstrated that a defect of Rac1 signaling impairs the postnatal development of renal papilla, leading to dysplasia without hydronephrosis. To the best of our knowledge, no clinical data showing an association between CAKUT and genetic mutations in Rac1 or related molecules have yet been reported, presumably because systemic dysfunction of Rac1 could lead to lethality at an early embryonic stage^76^. However, the present results suggest that some conditions or exposure to chemicals causing dysfunction of Rac1 or osmotic signaling in the perinatal period could potentially be the cause of some CAKUT cases for which the cause is unknown.

In conclusion, our study demonstrated that Rac1 in distal tubules plays an essential role in the postnatal development of renal papilla, and suggested a potential link between Rac1 and the NFAT5-dependent osmoprotective response. These findings revealed a new role of Rac1 in the development of the kidney in addition to other tissues.

## Supplementary Information

Supplementary Information 1.

Supplementary Information 2.

## Data Availability

The datasets used and/or analyzed during the current study available from the corresponding author on reasonable request.
