# Supplementary material for: Rac1 deficiency impairs postnatal development of the renal papilla
Source: Sci Rep. 2022 Nov 24;12:20310. doi: 10.1038/s41598-022-24462-5 (PMC9700760; doi:10.1038/s41598-022-24462-5)
Supplement: Supplementary file 1 — Supplementary Information 1. [file 41598_2022_24462_MOESM1_ESM.pdf]

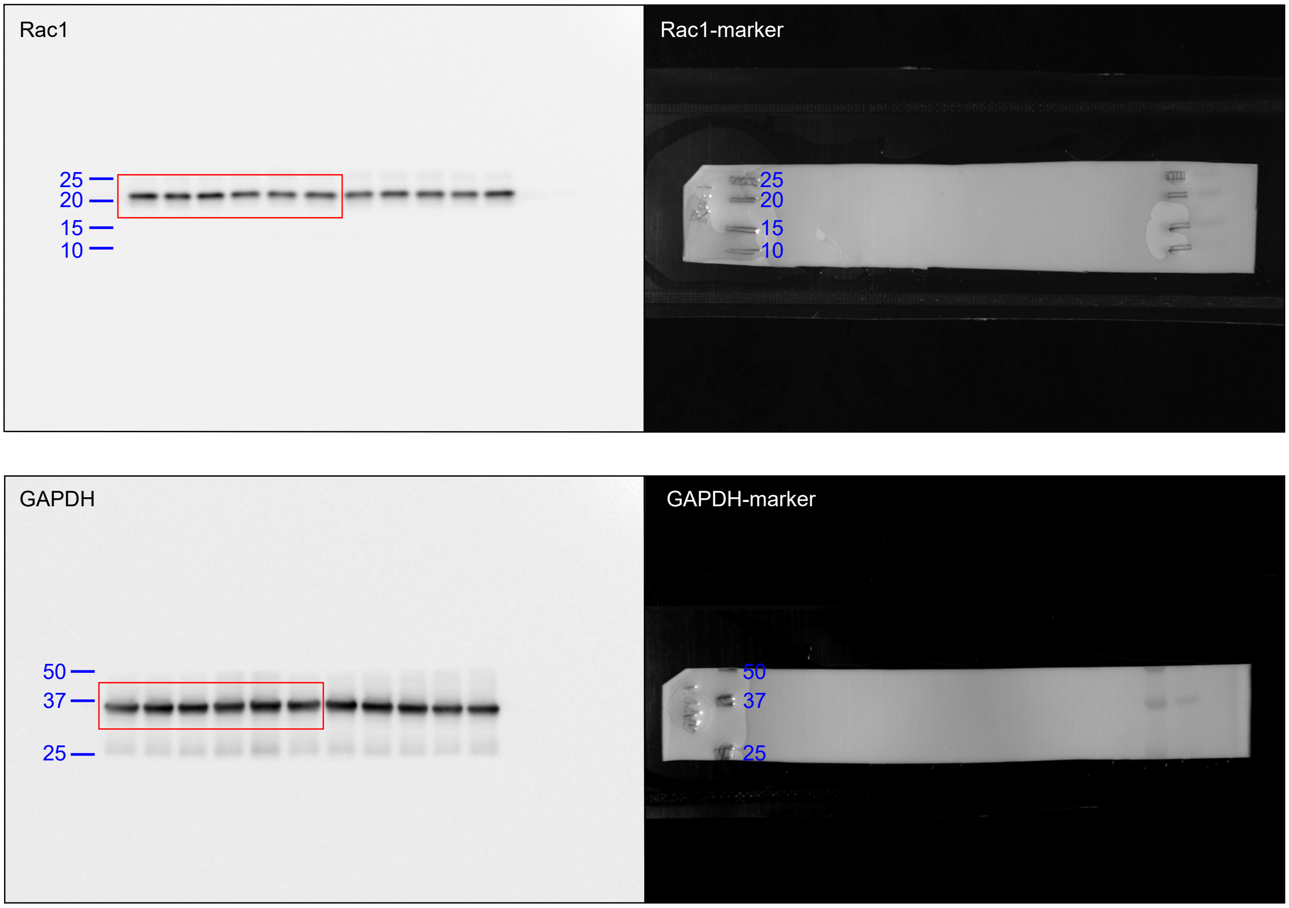

Uncropped western blot images of Figure 1b.

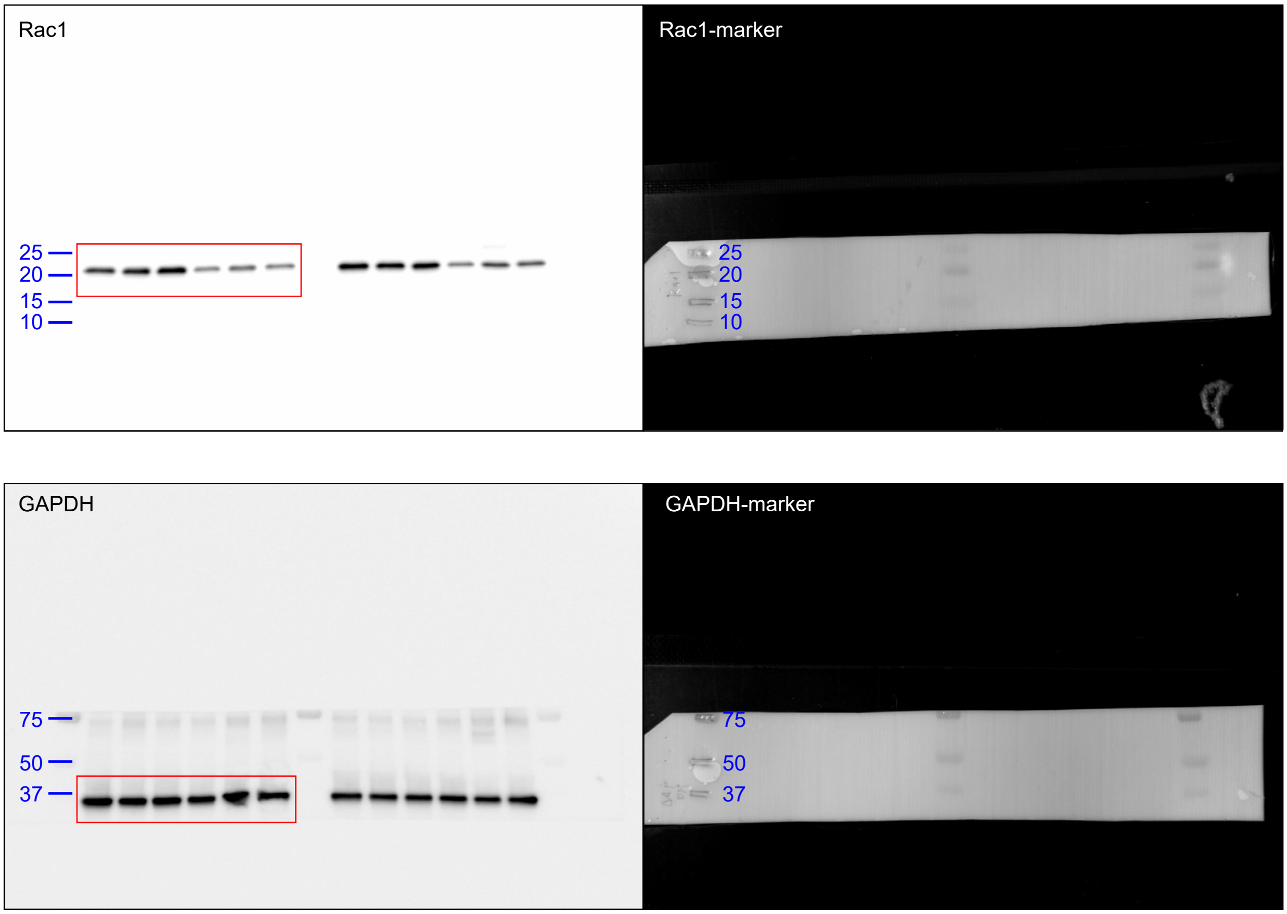

Uncropped western blot images of Figure 1c.

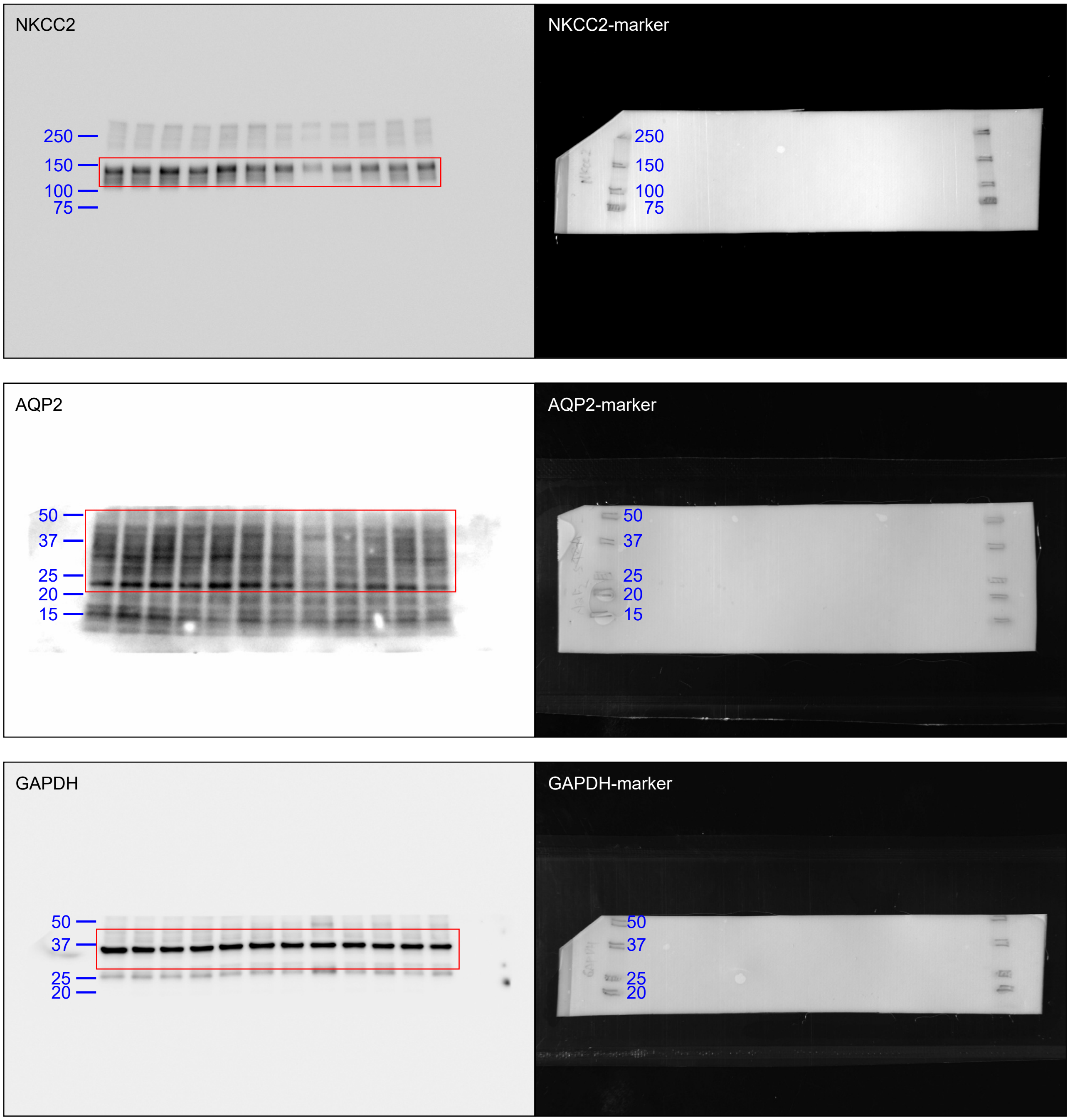

Uncropped western blot images of Figure 3a.

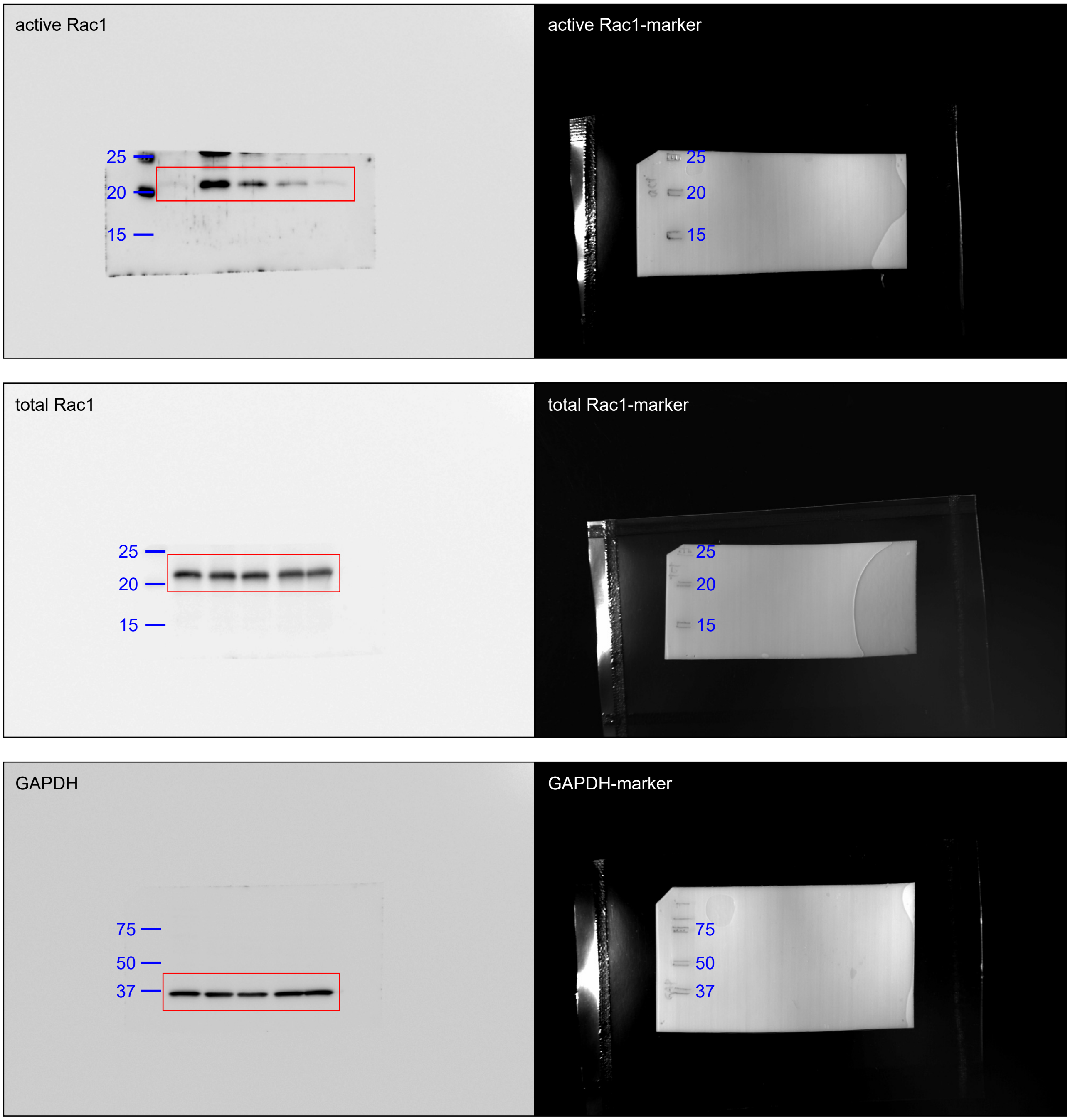

Uncropped western blot images of Figure 5a.

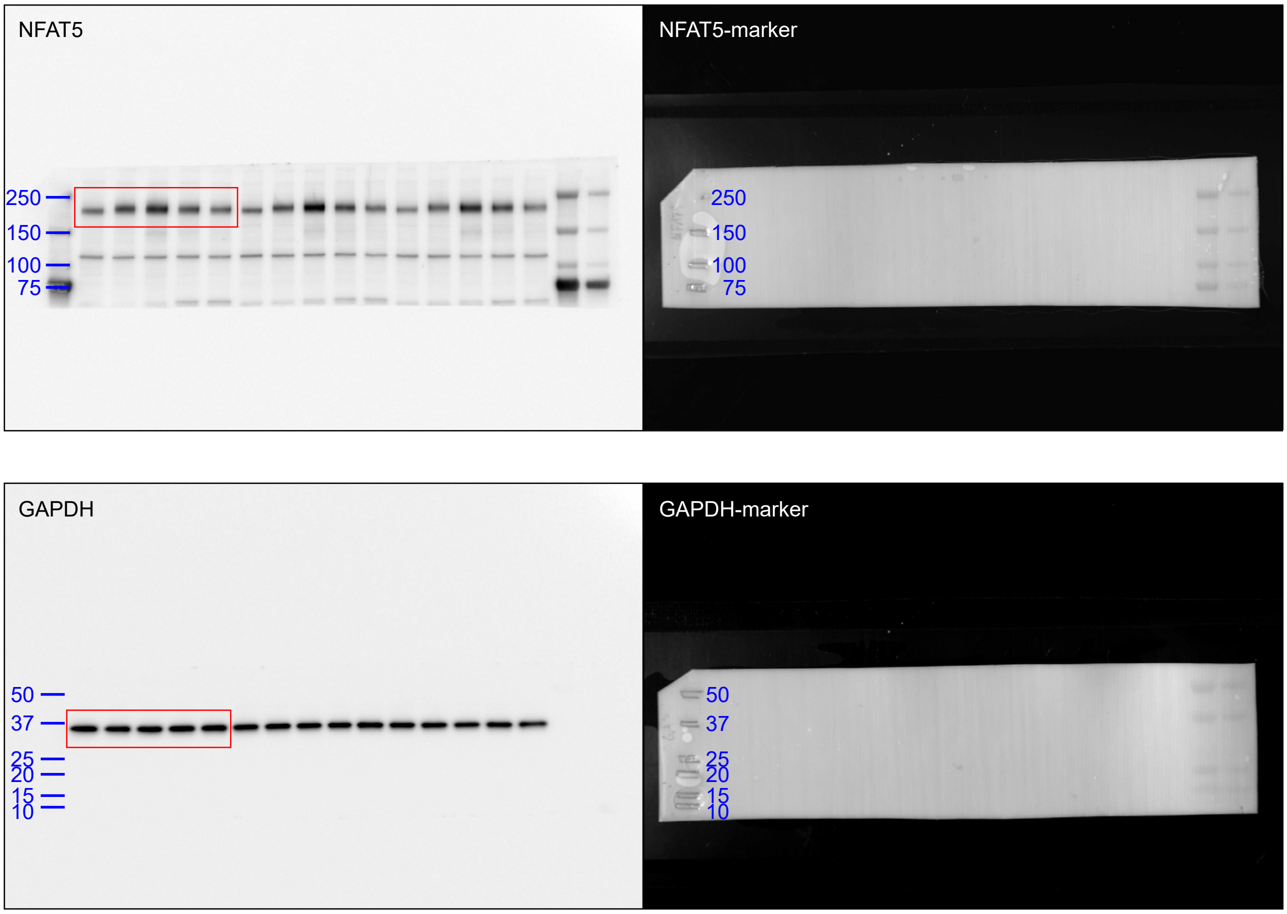

Uncropped western blot images of Figure 5b.

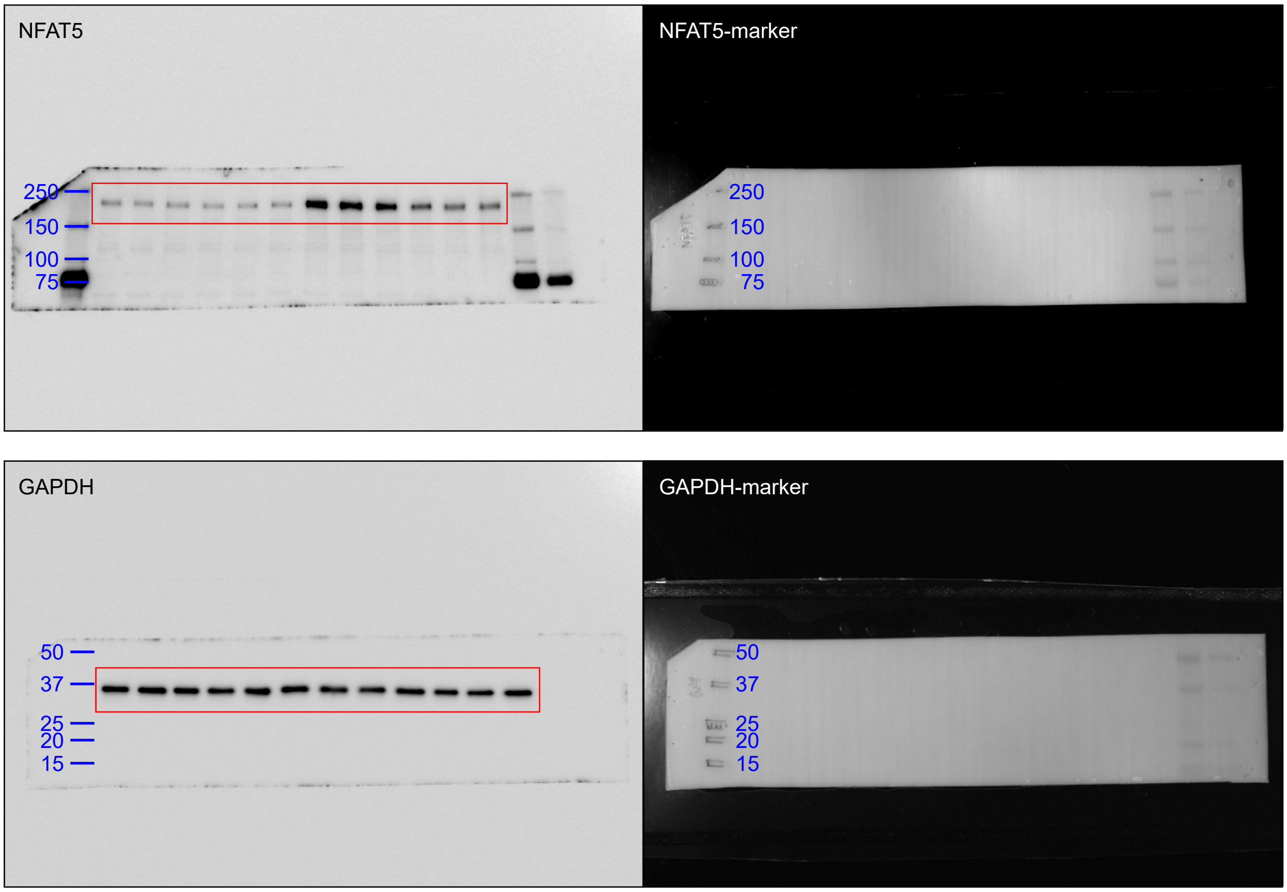

Uncropped western blot images of Figure 5d.

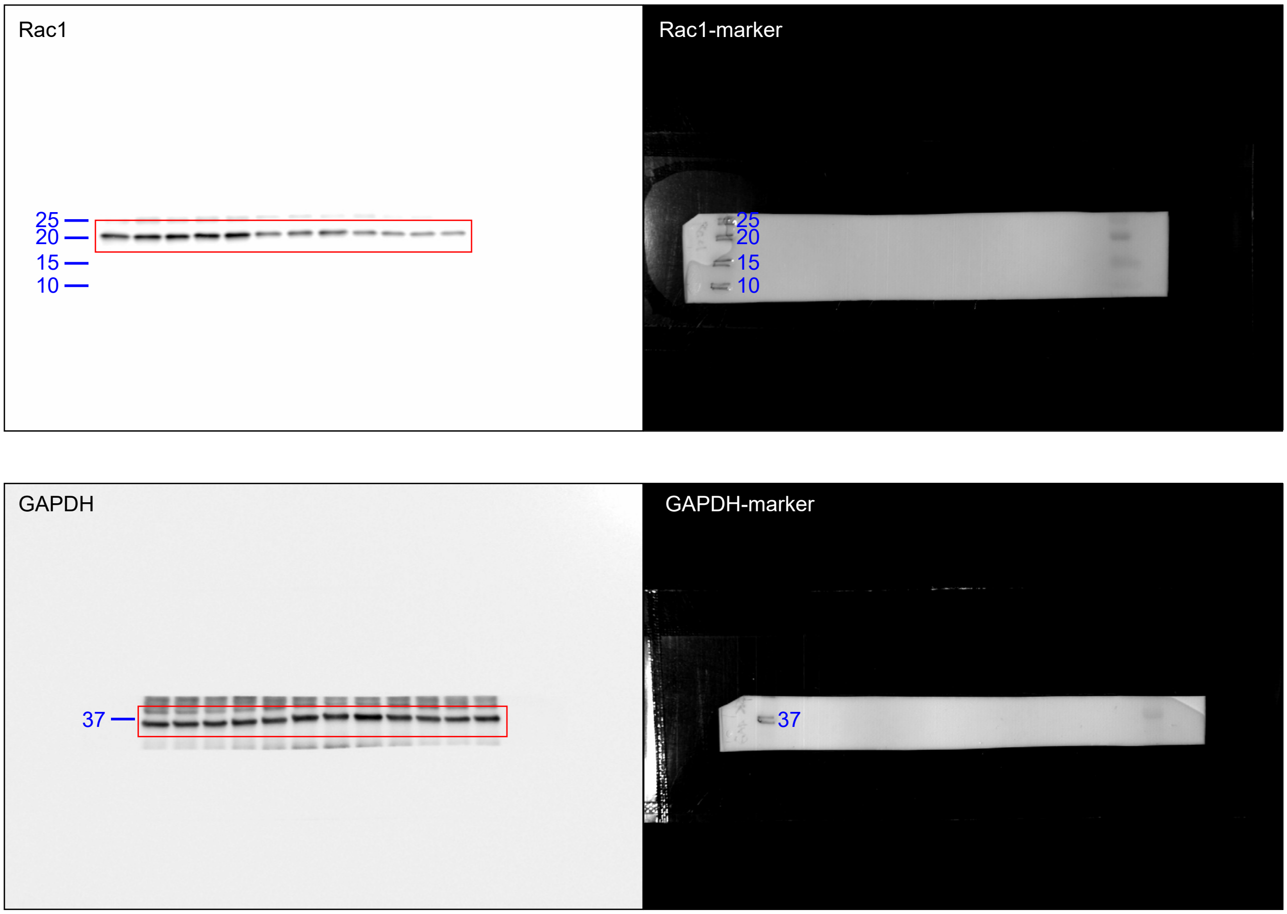

Uncropped western blot images of Supplementary Figure S1b.

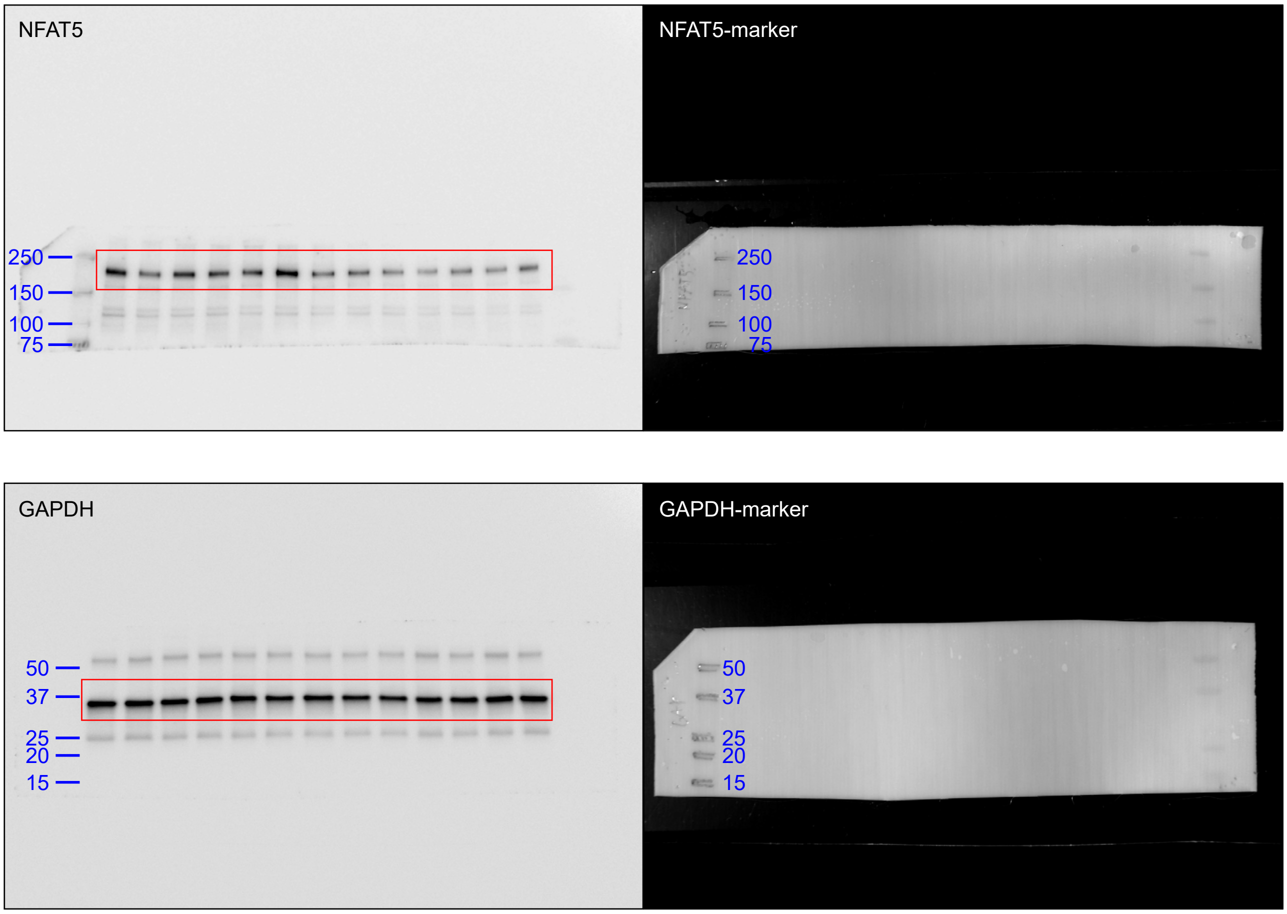

Uncropped western blot images of Figure 6a.

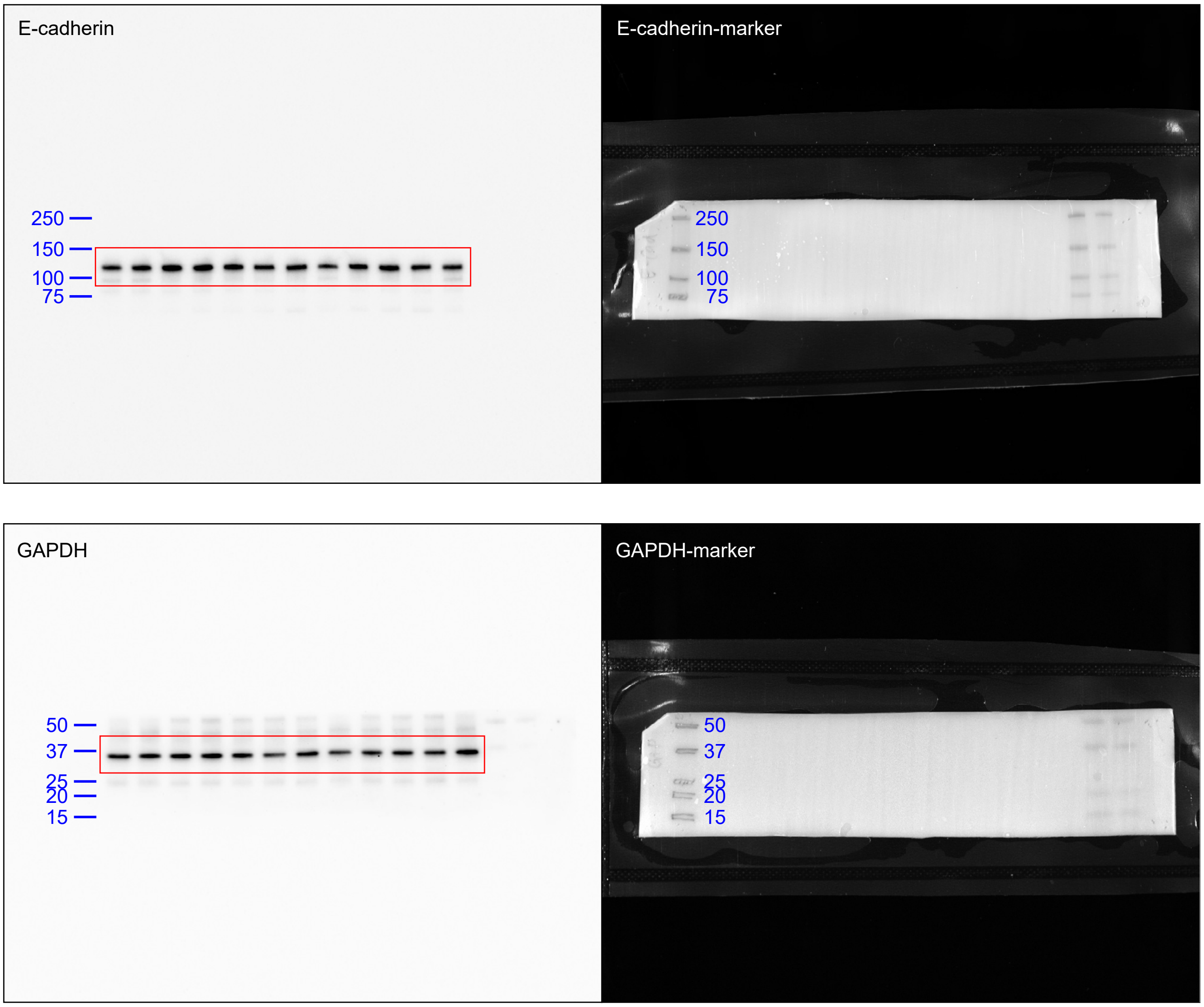

Uncropped western blot images of Figure 7c.

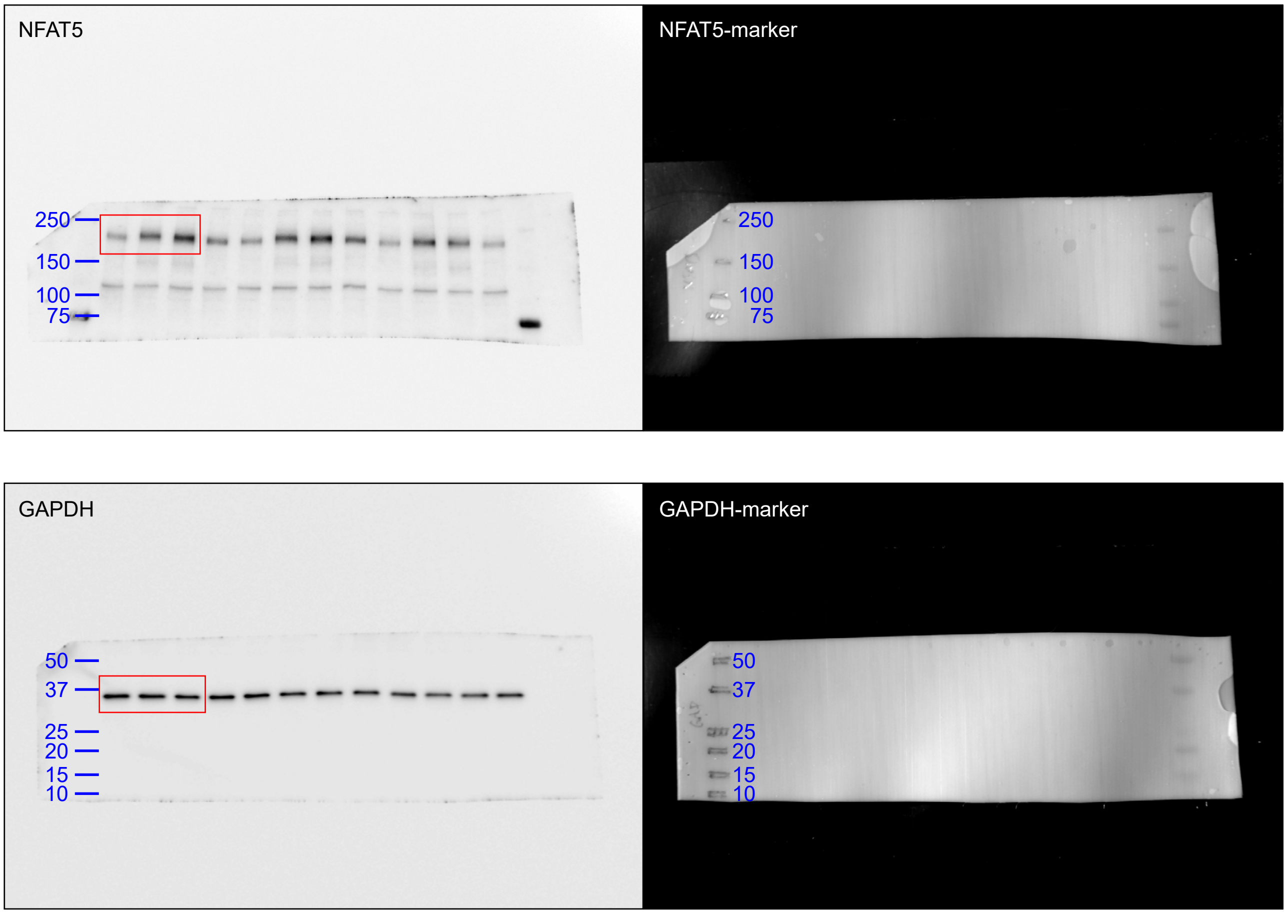

Uncropped western blot images of Supplementary Figure S3a.

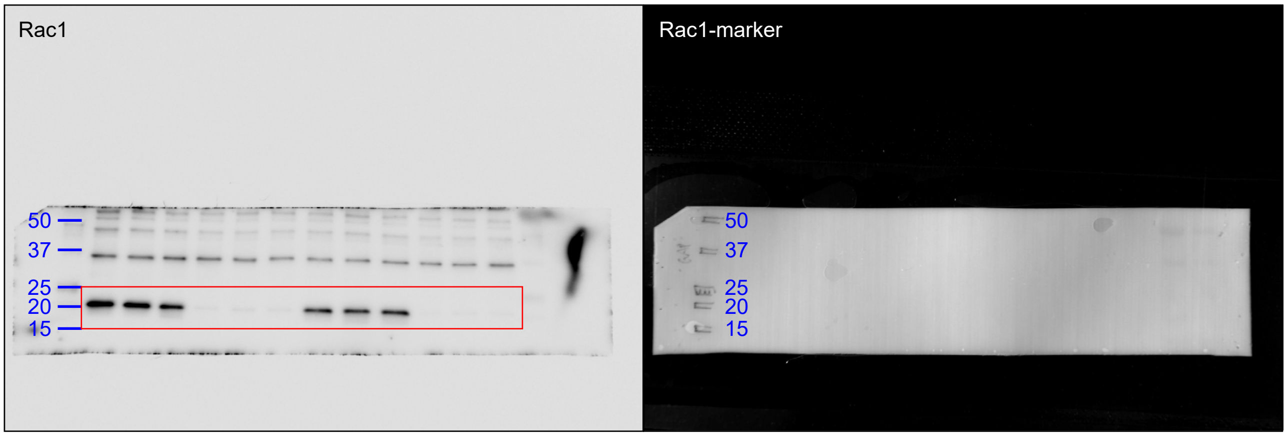

Uncropped western blot images of Supplementary Figure S3b.

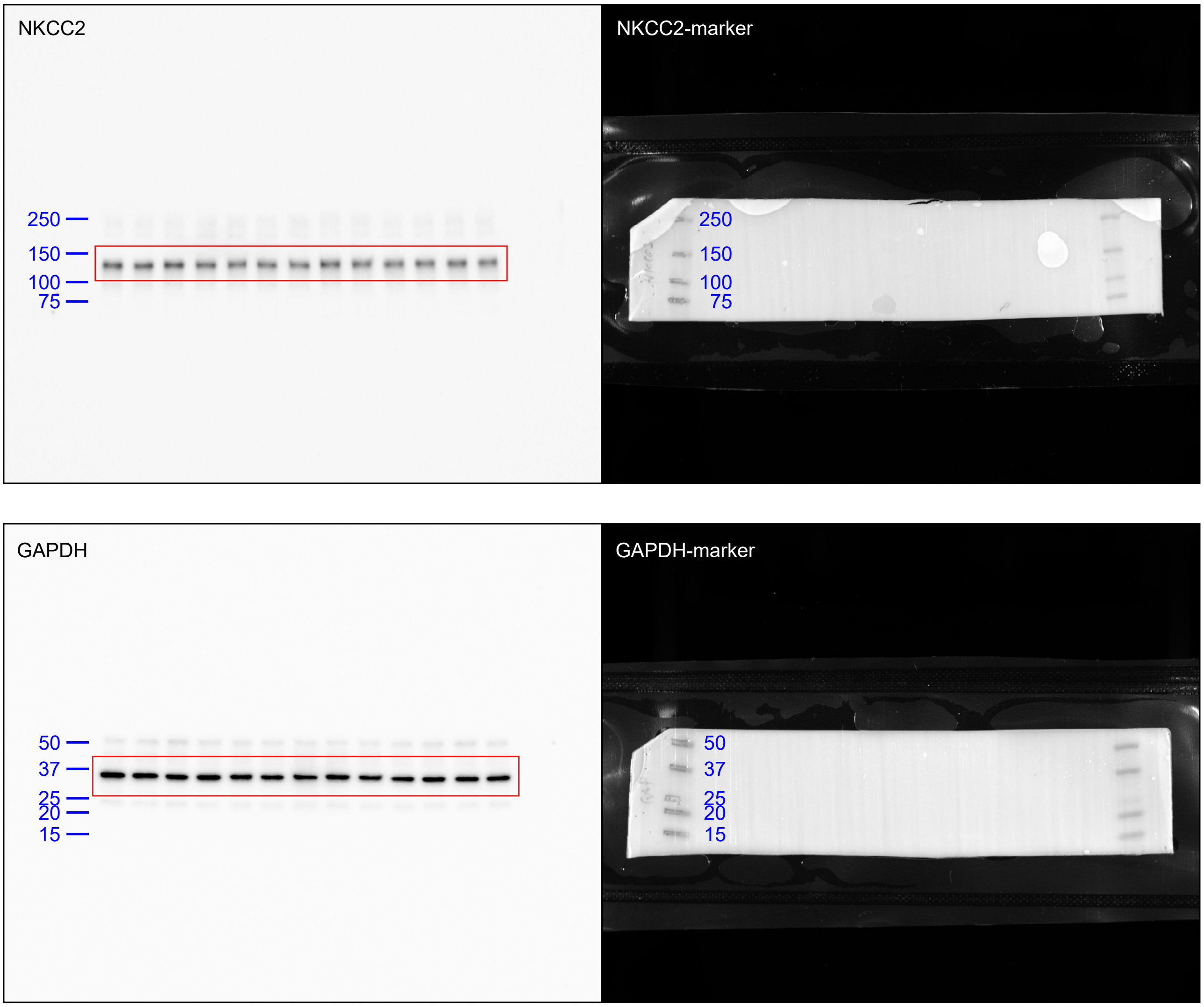

Uncropped western blot images of Figure S4.
