# Supplementary material for: Rac1 deficiency impairs postnatal development of the renal papilla
Source: Sci Rep. 2022 Nov 24;12:20310. doi: 10.1038/s41598-022-24462-5 (PMC9700760; doi:10.1038/s41598-022-24462-5)
Supplement: Supplementary file 2 — Supplementary Information 2. [file 41598_2022_24462_MOESM2_ESM.pdf]

Supplementary Information

Rac1 deficiency impairs postnatal development of the renal papilla

Nobuhiro Ayuzawa[1,*], Mitsuhiro Nishimoto[1,2], Wakako Kawarazaki[1], Shigeyoshi Oba[1], Takeshi Marumo[1,3], Atsu Aiba[4], & Toshiro Fujita[1,5,6,*]

[1]Division of Clinical Epigenetics, Research Center for Advanced Science and Technology, The University of Tokyo, Tokyo, Japan
[2]Department of Internal Medicine, Division of Nephrology and Hypertension, International University of Health and Welfare Mita Hospital, Tokyo, Japan
[3]Center for Basic Medical Research at Narita Campus, International University of Health and Welfare, Chiba, Japan
[4]Laboratory of Animal Resources, Center for Disease Biology and Integrative Medicine, Graduate School of Medicine, The University of Tokyo, Tokyo, Japan
[5]Shinshu University School of Medicine, Nagano, Japan
[6]Research Center for Social Systems, Shinshu University, Nagano, Japan
*Corresponding authors: ayuzawa-tky@umin.ac.jp, and Toshiro.FUJITA@rcast.u-tokyo.ac.jp

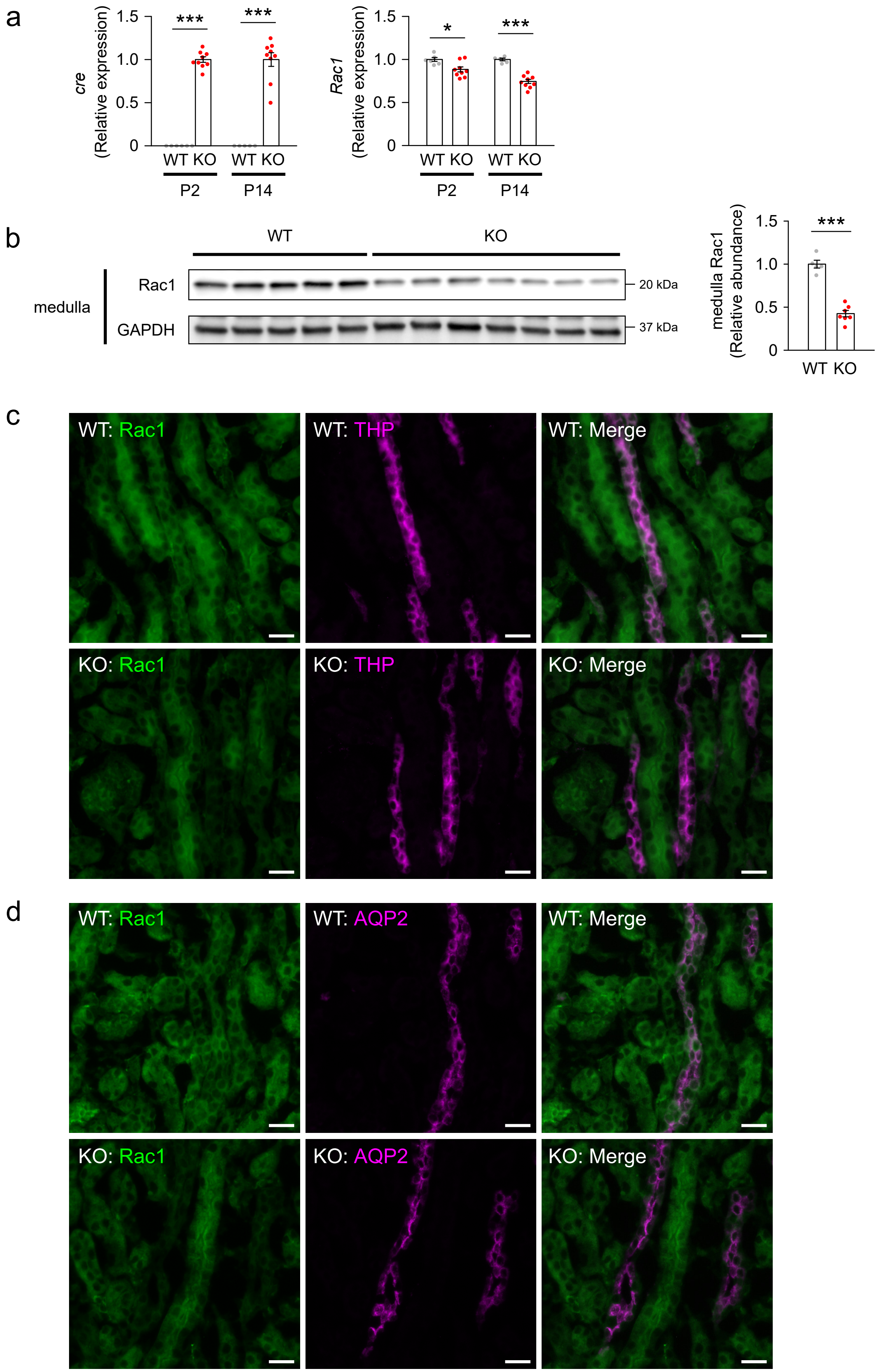

a
cre (Relative expression)
1.5
1.0
0.5
0
***
***
WT KO
WT KO
P2
P14
Rac1 (Relative expression)
*
***
b
WT
KO
medulla
Rac1
GAPDH
20 kDa
37 kDa
medulla Rac1 (Relative abundance)
c
WT: Rac1
WT: THP
WT: Merge
KO: Rac1
KO: THP
KO: Merge
d
WT: Rac1
WT: AQP2
WT: Merge
KO: Rac1
KO: AQP2
KO: Merge

Supplementary Figure S1. Rac1 expression in the kidneys of WT and KO mice at P2 and P14. (**a**) Quantitative analysis of *cre* and *Rac1* gene expression in the whole kidney samples at P2 and P14 (n=5–9 per group). The expression was normalized to a reference gene (*Rps29*) and reported relative to the KO group (*cre*) or WT group (*Rac1*) in each time point. (**b**) Immunoblots and quantities of Rac1 in the renal medulla at P14 (n=5–7 per group). The expression was normalized to a loading control (GAPDH) and reported relative to the WT group. (**c**,**d**) Immunofluorescent staining of Rac1 (green) with counterstaining of Tamm-Horsfall protein (THP) (magenta) for thick ascending limbs of loop of Henle (**c**) and AQP2 (magenta) for collecting ducts (**d**) in the kidneys at P14. Rac1 staining yielded negative results in the THP- and AQP2-positive tubules in KO mice. Scale bars, 20 μm. Data are expressed as mean ± SEM. Statistical significance was analyzed by unpaired t test. *P<0.05; ***P<0.001.

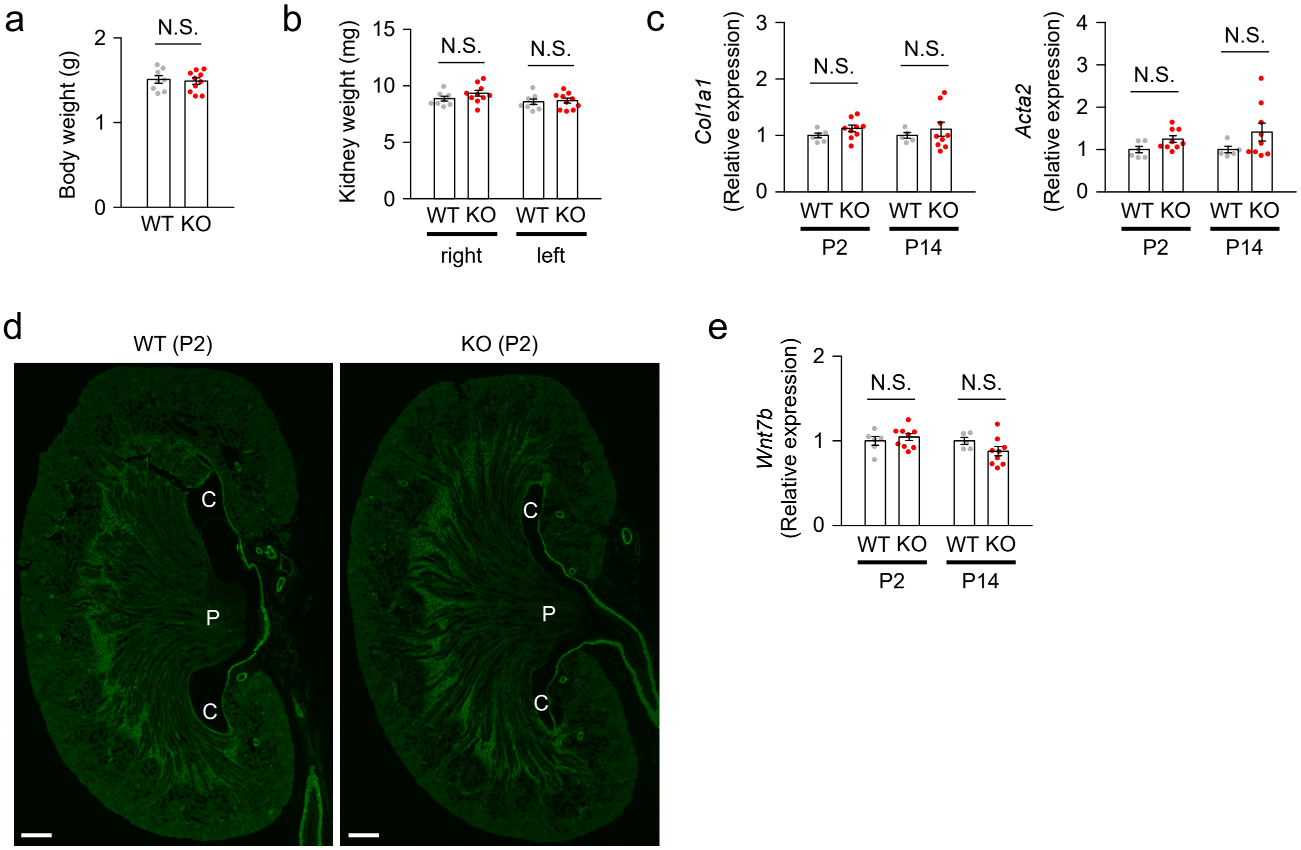

Supplementary Figure S2. Analysis of WT and KO mice at P2 and P14. (**a**) Body weight and (**b**) renal weight of WT and KO mice at P2 (*n*=8–10 per group). (**c**) Quantitative analysis of *Col1a1* and *Acta2* gene expression in the kidneys at P2 and P14 (*n*=5–9 per group). The expression was normalized to a reference gene (*Rps29*) and reported relative to the WT group in each time point. (**d**) Immunofluorescent staining of α-SMA (green) in the coronal sections of the kidneys at P2. P: papilla, C: calyx. No obvious differences in the staining pattern of α-SMA were found between WT and KO mice at P2. Scale bars, 200 μm. (**e**) Quantitative analysis of *Wnt7b* gene expression in the kidneys at P2 and P14 (*n*=5–9 per group). The expression was normalized to a reference gene (*Rps29*) and reported relative to the WT group in each time point. Data are expressed as mean ± SEM. Statistical significance was analyzed by unpaired *t* test. N.S.: not significant.

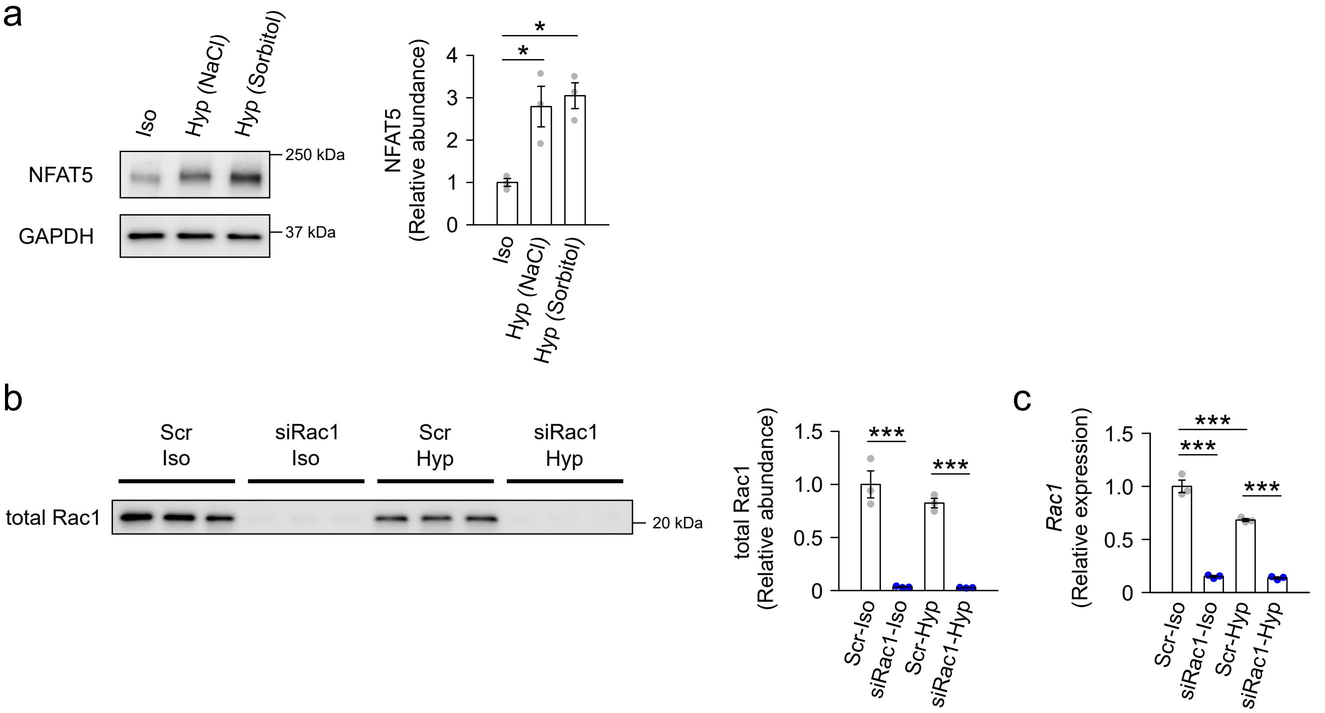

Supplementary Figure S3. Analysis of the effect of hyperosmotic stress in mIMCD3 cells. (**a**) Raising medium osmolarity by adding either NaCl or sorbitol increases expression of NFAT5 protein in mIMCD3 cells. mIMCD3 cells were cultured in either isotonic medium (Iso) or hyperosmotic medium (Hyp), which was prepared by adding NaCl or sorbitol, for 8 h. Representative immunoblots and quantities of NFAT5 are shown (n=3 per group). The expression was normalized to a loading control (GAPDH) and reported relative to the Iso group. (**b,c**) Efficacy of Rac1 knockdown by siRNA (related to Figure 5d,e, respectively). (**b**) Expression of total Rac1 protein was highly reduced in mIMCD3 cells treated with *Rac1* siRNA (siRac1) compared with that treated with scrambled siRNA (Scr), either after 8 hours of iso-osmotic or hyperosmotic treatment. (**c**) Expression of *Rac1* gene was highly reduced in mIMCD3 cells treated with siRNA targeting *Rac1* (siRac1) compared with that treated with scrambled siRNA (Scr), either after 16 hours of iso-osmotic or hyperosmotic treatment. Data are expressed as mean ± SEM. Statistical significance was analyzed by one-way ANOVA with Tukey's HSD post hoc test. *P<0.05; ***P<0.001.

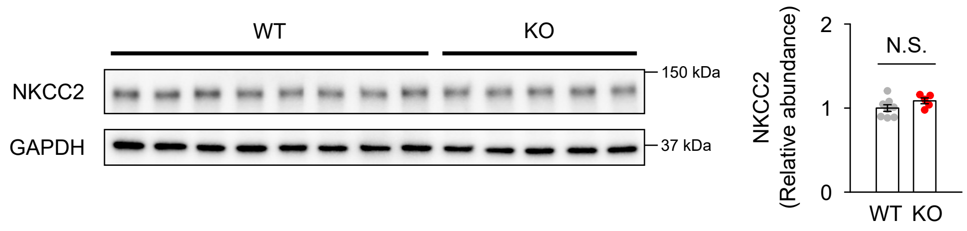

Supplementary Figure S4. Expression of NKCC2 in the kidneys of WT and KO mice at P2. Immunoblots and quantities of NKCC2 in the kidneys of WT and KO mice at P2 (n=5–8 per group). The expression was normalized to a loading control (GAPDH) and reported relative to the WT group. Data are expressed as mean ± SEM. Statistical significance was analyzed by unpaired *t* test.

Supplementary Table S1. Primer pairs used for quantitative RT-PCR.

Gene	Primer Pair (5′–3′)	
Col1a1	Forward	CTGACGCATGGCCAAGAAGACA
	Reverse	AAGCATACCTCGGGTTTCCACG
Acta2	Forward	CACGGCATCATCACCAACTG
	Reverse	GGCCACACGAAGCTCGTTAT
*Nfat*5	Forward	TGCTTTCTCAGCTTACCACGG
	Reverse	GTCCGCACAACATAGGGCTC
Akr1b3	Forward	ATCTGGAACTCAACAACGGC
	Reverse	GCGGTACCCCAAGTCAATAG
Slc6a12	Forward	GGTCCCTGAGGAAGGAGAGAT
	Reverse	GGGGATGAAGAAAGCTCCACC
Rps29	Forward	GGAGTCACCCACGGAAGTT
	Reverse	ATGAAGCCTATGTCCTTCGC
Wnt7b	Forward	CGCTACGGCATCGACTTTTCT
	Reverse	TCTGCCCGCCTCATTGTTG
